# Genomic and clinical determinants of response to Azacitidine plus venetoclax in acute myeloid leukemia: Results from the HM-SCREEN-Japan 02 study

**DOI:** 10.1371/journal.pone.0357898

**Published:** 2026-10-01

**Authors:** Tomoaki Ueda, Kentaro Fukushima, Sunggi Chi, Hiroshi Haeno, Goichi Yoshimoto, Hironori Arai, Daisuke Ikeda, Motoshi Ichikawa, Naoto Takahashi, Naoko Hosono, Takahiro Yamauchi, Takeshi Kondo, Shigeru Kusumoto, Junya Kuroda, Yosuke Minami

**Affiliations:** 1 Department of Hematology and Oncology, The University of Osaka Graduate School of Medicine, Suita, Osaka, Japan; 2 Department of Hematology, National Cancer Center Hospital East, Kashiwa, Chiba, Japan; 3 Division of Integrated Research Research Institute for Biomedical Sciences, Tokyo University of Science, Noda, Chiba, Japan; 4 Department of Hematology, Saga-ken Medical Centre Koseikan, Kasemachi, Saga, Japan; 5 Division of Hematology, Japanese Red Cross Narita Hospital, Narita, Chiba, Japan; 6 Department of Hematology, Oncology and Respiratory Medicine, Okayama University Graduate School of Medicine, Dentistry and Pharmaceutical Sciences, Kita-ku, Okayama, Japan; 7 Department of Hematology, NTT Medical Center Tokyo, Shinagawa-ku, Tokyo, Japan; 8 Department of Hematology, Nephrology and Rheumatology, Akita University Graduate School of Medicine, Hondo, Akita, Japan; 9 Department of Hematology and Oncology, University of Fukui, Yoshida-gun, Fukui, Japan; 10 Blood Disorders Center, Aiiku Hospital, Sapporo, Hokkaido, Japan; 11 Department of Hematology and Cell Therapy, Aichi Cancer Center, Chikusa-ku, Nagoya, Japan; 12 Division of Hematology and Oncology, Kyoto Prefectural University of Medicine, Kamigyo-ku, Kyoto, Japan; European Institute of Oncology, ITALY

## Abstract

The combination of azacitidine and venetoclax (Aza/Ven) is an effective treatment for acute myeloid leukemia (AML); however, biological factors underlying heterogeneous treatment responses across diverse genetic backgrounds remain incompletely defined. We analyzed 97 AML patients treated with Aza/Ven in the prospective HM-SCREEN-Japan 02 study using targeted next-generation sequencing (NGS) of 53 genes, cytogenetic analyses, and clinical variables. Somatic mutations were categorized into type 1 (*FLT3, PTPN11, WT1, IDH1/2, NPM1, NRAS*) and type 2 (*GATA2, KRAS, TP53, RUNX1, STAG2, ASXL1, ZRSR2, TET2*) groups based on a previously proposed framework describing clonal characteristics. Complete remission (CR) or CR with incomplete hematologic recovery (CRi) was achieved in 53.8%, 52.1%, and 50.0% of patients treated in the first-, second-, and ≥third-line settings, respectively. Among 53 first-line patients with pre-treatment NGS data, type 1–only mutations were more frequently observed in responders (CR/CRi 72%), whereas type 2–only mutations were enriched in non-responders (CR/CRi 33%). In the first-line setting, achievement of CR/CRi following Aza/Ven was associated with improved overall survival. In addition, an exploratory mathematical model integrating baseline clinical variables demonstrated strong discriminatory performance for treatment response, including in cases without established prognostic mutations. In conclusion, Aza/Ven demonstrated consistent clinical activity across treatment lines in this real-world cohort. Mutational patterns classified by a type 1/type 2 framework were associated with differential response patterns in the first-line setting. Integrative modeling approaches may support the interpretation of response heterogeneity, particularly in genetically uninformative AML.

## Introduction

Azacitidine is a hypomethylating agent that inhibits DNA methyltransferase, leading to DNA hypomethylation and reactivation of tumor suppressor genes. Monotherapy with azacitidine has been reported to provide a greater survival benefit than conventional therapies in elderly patients with acute myeloid leukemia (AML) [1,2]. Venetoclax targets the B-cell lymphoma 2 (BCL-2) protein and promotes cancer cell death in AML [3]. Azacitidine/venetoclax combination therapy (Aza/Ven) has been shown to be effective in treating newly diagnosed AML in elderly patients or those ineligible for intensive chemotherapy, with an overall response rate of 66.4% and a survival advantage over azacitidine monotherapy [4]. It has been reported that Aza/Ven targets leukemia stem cells through the suppression of amino acid metabolism [5,6]. Owing to its mechanism of action and favorable safety profile compared with conventional chemotherapy, this regimen has also been explored in transplant-eligible younger patients, particularly those with adverse-risk disease [7,8].

Makishima et al. proposed a mutation-based classification framework for myelodysplastic syndromes (MDS) and secondary AML, categorizing recurrent genetic alterations into type 1 mutations, which are mainly associated with signaling activation and disease progression, and type 2 mutations, which are related to epigenetic regulation and clonal hematopoiesis [9]. More recently, clonal evolution studies, including those by Winters et al., have suggested that Aza/Ven preferentially suppresses driver mutations acquired during the progression from MDS to AML, whereas antecedent MDS-related or pre-leukemic mutations frequently persist during treatment [10]. Together, these observations provide a biological rationale to hypothesize that the efficacy of Aza/Ven in AML may be influenced by the relative contribution of type 1 and type 2 mutations within the leukemic clone.

HM-SCREEN-Japan 02 (HMS02, UMIN-CTR UMIN000046371) is a Japanese multicenter prospective observational study evaluating the clinical utility of targeted sequencing. In the present study, we analyzed 97 AML patients who received Aza/Ven therapy within the HMS02 cohort and examined the association between gene mutation profiles and treatment outcomes, with a particular focus on type 1 and type 2 mutations. In addition, we performed exploratory supervised modeling using baseline clinical variables to characterize early treatment response, particularly in patients lacking clearly informative genomic predictors.

## Materials and methods

### Study design

Between 13/12/2021 and 31/03/2024, 188 patients with AML from 23 institutions across Japan were prospectively enrolled in the HMS02 study. Eligible patients were diagnosed with AML according to the 2017 World Health Organization (WHO) classification [11]. Bone marrow aspirates or peripheral blood samples containing at least 10% blasts were used for analysis and archived or stored specimens were not accepted. Further details on the inclusion criteria are available in our previous report [12]. Following submission, annotated genomic reports were returned to the participating institutions. Data were accessed for research purposes on 10/FEB/2024, when the clinical database was locked and the dataset was extracted for analysis. The study was conducted in accordance with the Declaration of Helsinki and approved by the institutional review board of each participating institution. Written informed consent was obtained from all patients included in this study.

### NGS analysis and data collection

We employed the AmoyDx® Myeloid Blood Cancer Panel, developed by Amoy Diagnostics Co., Ltd. (China). The AmoyDx® Myeloid Blood Cancer Panel is a genomic sequencing technology developed by Amoy that detects 53 genes using bone marrow fluid or peripheral blood [12].

Treatment decisions were at the discretion of the treating physicians. Clinical data including age, sex, treatment regimen, treatment response, and survival status were collected. In addition, information on cytogenetic karyotype, peripheral blood parameters (white blood cell count, hemoglobin level, and platelet count), and blast percentages in peripheral blood and bone marrow, peripheral blood *WT1* (Wilms tumor 1) mRNA expression at diagnosis was obtained. Flow cytometry was performed using standardized instruments at each participating institution or clinical testing company. The intensity of marker expression, including CD7 and CD56, was classified as negative, dim, or positive based on the judgment of the treating physicians at each institution.

### Predictive modeling and validation

We developed supervised classification models to characterize early response to Aza/Ven. The primary endpoint for modeling was a binary response after the first cycle (complete remission (CR)/ CR with incomplete hematologic recovery (CRi) vs non-CR), with CR/CRi designated as the positive class.

Baseline clinical variables available before Aza/Ven were considered: age (years), sex, white blood cell count (WBC, /µL), peripheral blood (PB) blast percentage, bone-marrow nucleated cell count (BM-NCC, /µL), bone-marrow (BM) blast percentage, hemoglobin (g/dL), platelet count (×10^4^/µL), PB *WT1* mRNA level (log10 copies/µg RNA), and immunophenotype markers CD7 and CD56.

We first constructed an analysis dataset comprising baseline clinical and laboratory variables (age, sex, white blood cell count, peripheral blood and bone marrow blast percentages, bone marrow nucleated cell count, peripheral blood *WT1* expression, hemoglobin, platelet count, and expression of CD7 and CD56) together with the clinical response endpoint (CR/CRi vs non-CR). The response variable was coded as a binary factor with “non-CR” as the reference (negative) class and “CR/CRi” as the positive class. Sex was encoded as a binary factor (female/male), and CD7 and CD56 were treated as ordered categorical variables with three levels (NEGATIVE < dim < POSITIVE).

All preprocessing steps were implemented using the recipes framework in R and were applied identically to all models. The raw peripheral blood *WT1* copy number was discarded, and only its base-10 logarithm was retained as a predictor. Missing values in categorical predictors (sex, CD7, CD56) were imputed using the most frequent category (mode), and missing values in continuous predictors were imputed using the median of the training data. Ordered categorical variables (CD7 and CD56) were converted to numeric scores (1, 2, and 3 for NEGATIVE, dim, and POSITIVE, respectively). All continuous predictors were then standardized to have mean 0 and unit variance (z-score normalization) based on the training set, and the same centering and scaling parameters were applied to the corresponding variables in the test observations.

To evaluate model performance in the presence of a small sample size, we used leave-one-out cross-validation (LOOCV). For each iteration, one patient was held out as the test case, and the remaining n−1 patients were used as the training set. The entire preprocessing recipe (imputation, ordinal coding, and normalization) was refit within each training set and then applied to both the training and held-out test observation to prevent information leakage.

On each LOOCV training set, we fitted (i) a linear discriminant analysis (LDA) model using MASS::lda and (ii) a linear support vector machine (SVM) using e1071::svm. For LDA, we used default priors equal to the empirical class proportions and a small tolerance parameter (tol = 1 × 10^-4^) to ensure numerical stability. For the SVM, we specified a linear kernel with cost parameter C=1, disabled additional scaling (because predictors had already been standardized), enabled probabilistic output, and used inverse class-frequency weights to mitigate class imbalance. For each held-out subject, the predicted class label and the predicted probability of CR/CRi were stored for both models.

After completion of leave-one-out cross-validation, predictions for all patients were pooled to construct a confusion matrix (reference category: non-CR). From this confusion matrix, we calculated overall accuracy, sensitivity, specificity, and positive and negative predictive values to summarize the classification performance of each model.

Receiver-operating characteristic (ROC) curves were generated using the cross-validated predicted probabilities for CR/CRi as decision scores, with CR/CRi defined as the positive class. Areas under the ROC curve (AUCs) and their 95% confidence intervals were estimated using DeLong’s method [13], and the AUCs of the LDA and SVM models were compared using DeLong’s test for correlated ROC curves.

For an exploratory cutoff-based analysis, the optimal cutoff value for the LOOCV-predicted LDA probability was determined using Youden’s index from the ROC curve. Cases were classified into predicted responder and predicted non-responder groups according to this cutoff. CR/CRi rates were compared between the two groups using Fisher’s exact test. OS probabilities were estimated using the Kaplan–Meier method and compared using the log-rank test.

For interpretability of the LDA model, we re-estimated the LDA coefficients using the entire dataset after applying the same preprocessing steps described above. The detailed explanation is provided in the Supplementary Methods. Analyses were performed in R using tidyverse, recipes, MASS, e1071, and pROC. The full analysis script, including the resampling protocol and LDA coefficient calculation on the preprocessed predictor scale, is provided in the accompanying code.

### Statistical analysis

Overall survival (OS) was calculated using the Kaplan-Meier method and compared using the 2-sided log-rank test. All reported *P* values are 2-sided, and differences were considered significant at *P* ≤ 0.05. OS was defined from the date of diagnosis until last follow-up or death. Comparisons among categorical variables and numerical variables were carried out using Fisher’s exact test and the Mann-Whitney *U* test, respectively. Statistical analyses were performed using EZR software [14].

## Results

### Patient characteristics

Of the 188 patients prospectively enrolled in the HMS02 study, 97 who received Aza/Ven therapy were included in the present analysis. The median age was 70 years, and the cohort comprised 61 males and 36 females (Table 1). According to the 2017 WHO classification, 46% of the cases were classified as AML with myelodysplasia-related changes (AML-MRC).

**Table 1:** Patient characteristics.

Characteristics	Aza/Ven (n = 97)	Chemotherapy (n = 122)
**Age, years, median**	70	57.5
< 65 years (%)	31 (32)	80 (66)
65 −74 years (%)	38 (39)	29 (24)
≥ 75 years (%)	28 (29)	13 (11)
**Sex (M/F), n (%)**	61 (63)/36 (37)	69 (57)/53 (43)
**WHO 2017 disease classification, n (%)**		
AML with myelodysplasia-related changes	45 (46)	21 (17)
AML with maturation	10 (10)	16 (13)
Therapy-related myeloid neoplasms	9 (9)	8 (7)
Acute monoblastic/monocytic leukemia	8 (8)	7 (6)
AML without maturation	6 (6)	14 (11)
AML with t(8;21)(q22;q22.1)	4 (4)	10 (8)
AML with mutated *NPM1*	3 (3)	11 (9)
AML with minimal differentiation	3 (3)	5 (4)
AML with inv(3)(q21.3q26.2) or t(3;3)(q21.3;q26.2)	3 (3)	3 (2)
AML with t(9;11)(p21.3;q23.3)	2 (2)	3 (2)
Acute myelomonocytic leukemia	2 (2)	5 (4)
AML with inv(16)(p13.1q22)	1 (1)	5 (4)
Pure erythroid leukemia	1 (1)	1 (1)
Acute promyelocytic leukemia with *PML-RARA*	0 (0)	8 (7)
Others	0 (0)	4 (3)
**WHO 2022 disease classification, n (%)**		
AML with myelodysplasia related	46 (47)	30 (25)
AML with *NPM1* mutation	13 (13)	15 (12)
Myeloid neoplasm post cytotoxic therapy	9 (9)	8 (7)
AML with maturation	5 (5)	9 (7)
Acute monoblastic/monocytic leukemia	5 (5)	4 (3)
AML without maturation	4 (4)	10 (8)
AML with *RUNX1::RUNX1T1* fusion	4 (4)	10 (8)
AML with *KMT2A* rearrangement	3 (3)	5 (4)
AML with *MECOM* rearrangement	3 (3)	3 (2)
AML with minimal differentiation	2 (2)	2 (2)
AML with *CBFB::MYH11* fusion	1 (1)	5 (4)
Acute myelomonocytic leukemia	1 (1)	3 (2)
Pure erythroid leukemia	1 (1)	1 (1)
Acute promyelocytic leukaemia with *PML::RARA* fusion	0	8 (7)
AML with *BCR::ABL1* fusion	0	1 (1)
Acute megakaryoblastic leukemia	0	1 (1)
Others	0	7 (6)
**Lab data at diagnosis, median (range)**		
White blood cells (10^3^/ul)	6.5 (0.2-284.6)	9.75 (0.2-336.8)
Hemoglobin (g/dl)	8.2 (3.1-16.6)	8.7 (2.9-16.6)
Platelet (10^4^/ul)	6.6 (0.4-77.3)	5.1 (0.3-55.8)
Peripheral blood blast (%)	16 (0-100)	45.6 (0-99)
Bone marrow blast (%)	42.1 (5.5-100)	66.2 (2.8-99)
Peripheral blood *WT1* mRNA (copy/ugRNA)	29000 (50-310000)	25500 (50-700000)
Lines of treatment*, n(%)		
1st	66 (68)	108 (65)
2nd	25 (26)	29 (17)
≥3rd	15 (15)	30 (18)
* Numbers represent treatment episodes; some patients received Aza/Ven in multiple lines.		
Allo-HSCT, n(%)	31 (32)	53 (43)

Allo-HSCT; allogeneic hematopoietic stem cell transplantation, AML; acute myeloid leukemia, Aza; azacitidine, Ven; venetoclax, *WT1*; Wilms tumor 1

With respect to treatment line, 66 patients received Aza/Ven as first-line therapy, 25 as second-line therapy, and 15 as third-line or later therapy; nine patients received Aza/Ven in more than one treatment line. Thirty-one patients (32%) subsequently underwent allogeneic hematopoietic stem cell transplantation.

### Clinical activity of Aza/Ven across treatment lines

The median follow-up duration for the entire Aza/Ven cohort (n = 97) was 12.49 months (range, 0.53–165.96), and the median overall survival (OS) was 16.0 months (Fig 1A). Patients received a median of 2 cycles of Aza/Ven treatment (range, 1–48). Among the 66 patients who received Aza/Ven as first-line therapy, 53.8% achieved CR/CRi, whereas 46.2% did not achieve CR/CRi (non-CR). Comparable response rates were observed in later treatment settings, with CR/CRi achieved in 52.1% of second-line cases and 50.0% of third-line or later cases (Fig 1B). In each treatment-line group, including first-line, second-line, and third-line or later therapy, the median number of cycles from Aza/Ven initiation to achievement of CR/CRi was 1 (range, 1–2).

**Fig 1:**
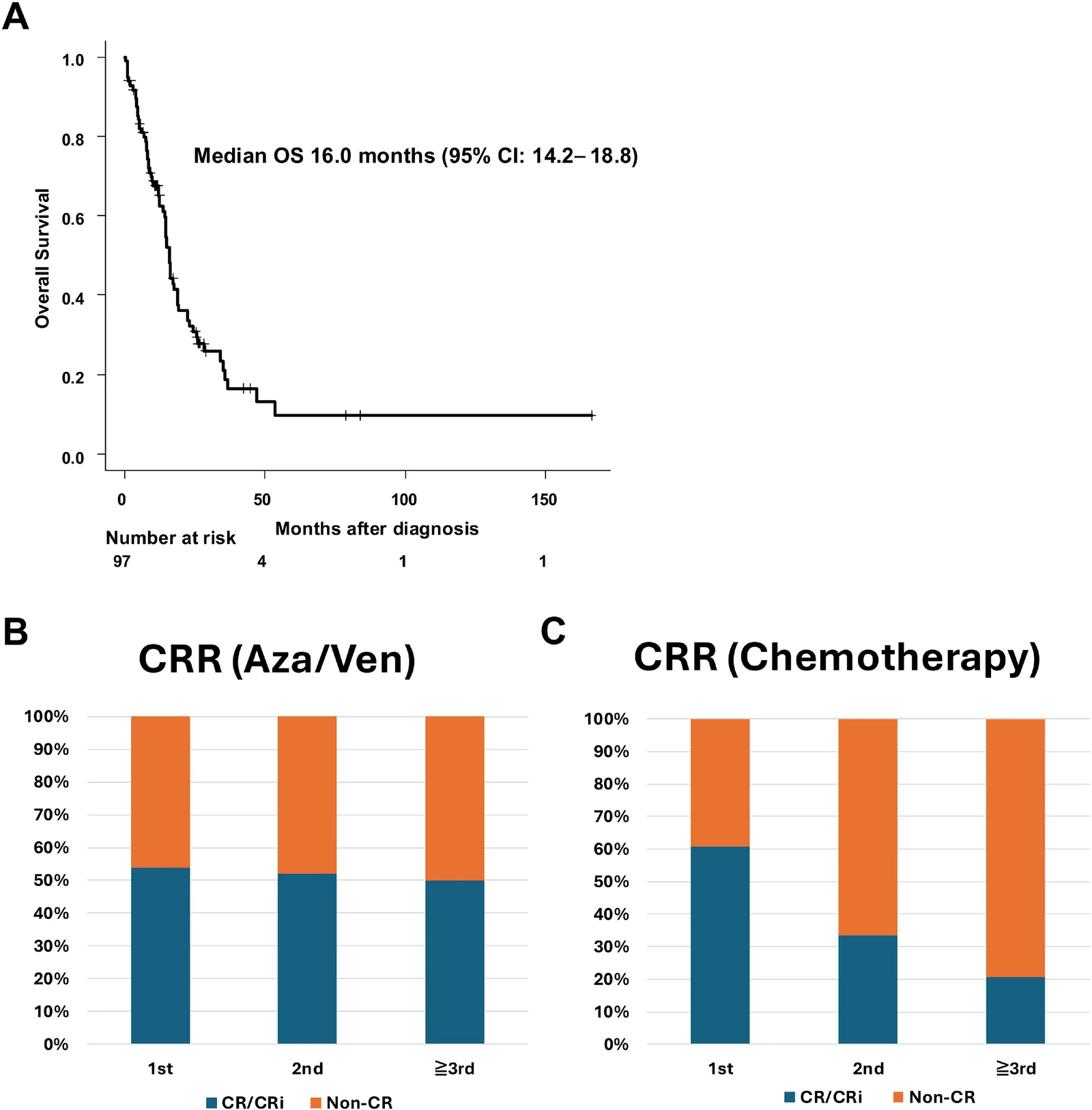
Overall survival and response rates of Aza/Ven across treatment lines. A. Overall survival (OS) of the entire Aza/Ven cohort (n  =  97). Median OS was 16.0 months (95% CI: 14.2–18.8). B. Complete remission rate (CRR) by line of therapy (Aza/Ven). 1st line (n  =  66): CR/CRi (n  =  35), non-CR (n  =  30), N/A (n  = 1). 2^nd^ line (n = 24): CR/CRi (n = 12), non-CR (n = 11), N/A (n = 1). 3^rd^ line and beyond (n = 14): CR/CRi (n = 7), non-CR (n = 7). C. CRR by line of therapy (Chemotherapy). 1^st^ line (n = 109): CR/CRi (n = 65), non-CR (n = 42), N/A (n = 2). 2^nd^ line (n = 29): CR/CRi (n = 9), non-CR (n = 18), N/A (n = 2). 3^rd^ line and beyond (n = 25): CR/CRi (n = 5), non-CR (n = 19), N/A (n = 1). Aza, azacitidine; CR, complete remission; CRi, CR with incomplete hematologic recovery; N/A, not available; Ven, venetoclax.

In contrast, among patients treated with conventional chemotherapy within the HMS02 cohort, CR/CRi rates declined with increasing treatment lines, from 60.7% in the first-line setting to 33.3% in the second-line and 20.8% in the third-line or later settings (Fig 1C).

### Association between first-line Aza/Ven response and overall survival

Among patients who received Aza/Ven as first-line therapy, we focused on 53 cases for which targeted next-generation sequencing (NGS) was performed immediately prior to treatment initiation. We first evaluated the impact of incorporating NGS data into prognostic classification. Of 30 patients initially classified as intermediate-risk based on cytogenetic abnormalities alone, 5 were reclassified as favorable risk and 9 as adverse risk according to the European LeukemiaNet (ELN) 2022 risk stratification (Fig 2A) [15].

**Fig 2:**
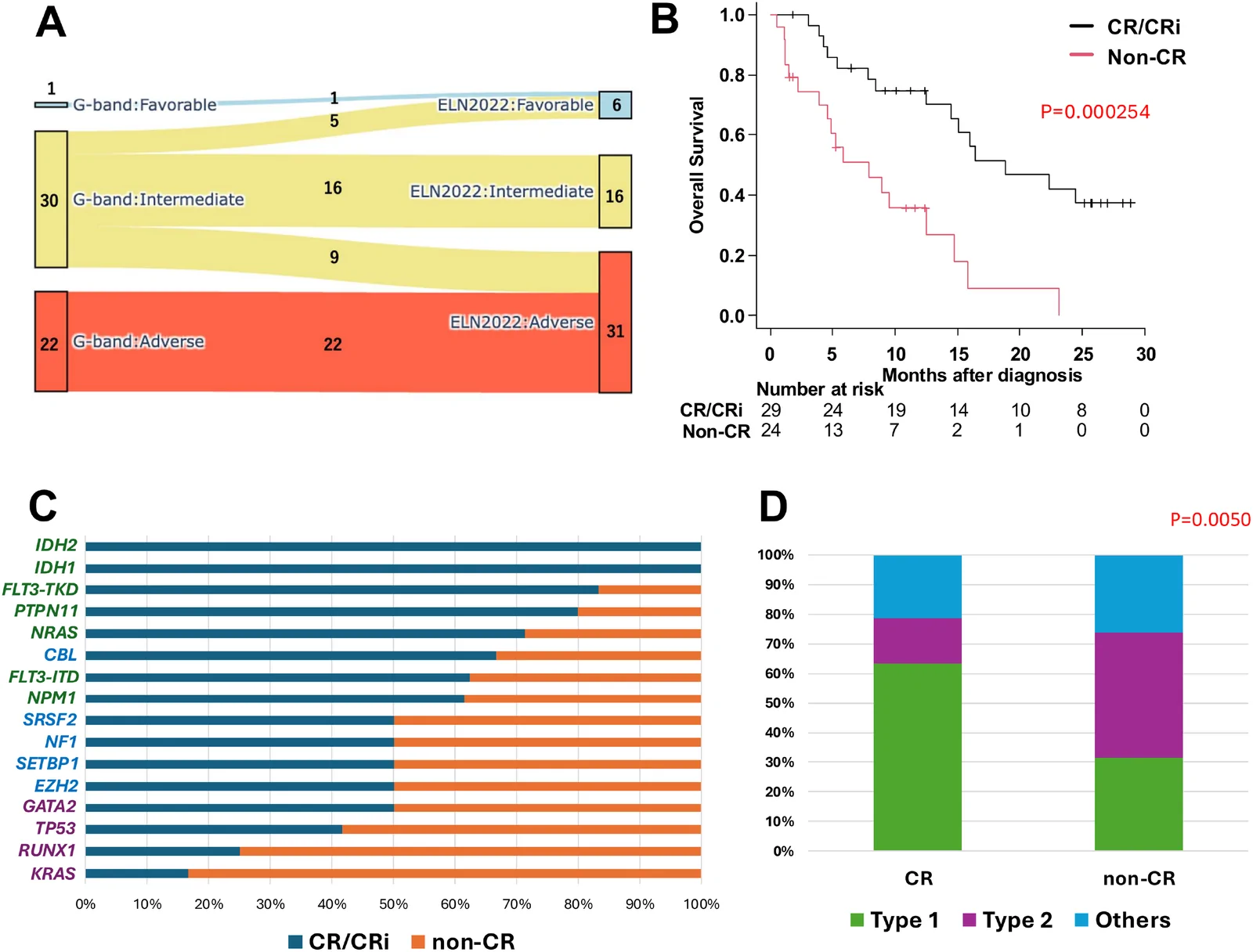
Mutation patterns and survival according to response to Aza/Ven. A. Concordance between G-banding and ELN 2022 cytogenetic risk classifications. Sankey diagram showing reclassification of patients (n = 53) from conventional G-banding–based cytogenetic risk groups (left) to ELN 2022 risk groups (right). Flow width is proportional to the number of cases. Of 1 patient classified as favorable by G-banding, 1 remained favorable under ELN 2022. Of 30 patients classified as intermediate, 16 remained intermediate, 5 were reclassified as favorable, and 9 were reclassified as adverse. Genes involved in risk reclassification included *NPM1* for intermediate-to-favorable reclassification (n = 5), and *EZH2* (n = 2), *RUNX1* (n = 2), *SRSF2* (n = 2), *TP53* (n = 2), and *U2AF1* (n = 1) for intermediate-to-adverse reclassification. All 22 patients classified as adverse remained adverse under ELN 2022, resulting in 31 adverse cases in total. B. Overall survival according to response to first-line Aza/Ven. C. Treatment response according to gene mutation (n ≥ 2). CR/CRi ratios were as follows: *IDH1* (100%, n = 3), *IDH2* (100%, n = 3), *FLT3-TKD* (83%, n = 6), *PTPN11* (80%, n = 5), *NRAS* (71%, n = 7), *CBL* (67%, n = 3), *FLT3-ITD* (63%, n = 8), *NPM1* (62%, n = 13), *SRSF2* (50%, n = 4), *EZH2* (50%, n = 2), *SETBP1* (50%, n = 2), *NF1* (50%, n = 4), *GATA2* (50%, n = 2), *TP53* (42%, n = 12), *RUNX1* (25%, n = 4), and *KRAS* (17%, n = 6). Green indicates Type 1 mutations, purple indicates Type 2 mutations, and blue indicates other mutations. D. Distribution of treatment response (CR/CRi vs non-CR). CR/CRi (n = 52): type 1 (63%, n = 33), type 2 (15%, n = 8), others (21%, n = 11). Non-CR (n = 38): type 1 (32%, n = 12), type 2 (42%, n = 16), others (26%, n = 10) (p = 0.0050). Aza, azacitidine; CR, complete remission; CRi, CR with incomplete hematologic recovery; ELN, European LeukemiaNet; Ven, venetoclax.

Following first-line Aza/Ven therapy, 29 patients (54.7%) achieved CR/CRi, and treatment response was significantly associated with OS (Fig 2B). Baseline characteristics of patients achieving CR/CRi and those with non-CR are summarized in Table 2. Baseline cytogenetic risk and ELN 2022 risk classification were not comparable between the two groups, whereas no other significant differences were observed, including classification according to the previously proposed Aza/Ven risk model [16,17].

**Table 2:** Patients’ characteristics.

Characteristics	CR/CRi (n = 29)	non-CR (n = 24)	p value
**Age, years, median**	70	71.5	0.899
< 65 years (%)	3 (10)	4 (17)	
65 −74 years (%)	18 (62)	11 (46)	
≥ 75 years (%)	8 (28)	9 (35)	
**Sex (M/F)**	20 (69) /9 (31)	12 (50)/ 12 (50)	0.259
**WHO 2017 disease classification, n(%)**			0.528
AML with myelodysplasia-related changes	16 (55)	16 (67)	
AML with maturation	4 (14)	2 (8)	
Therapy-related myeloid neoplasms	2 (7)	1 (4)	
Acute monoblastic/monocytic leukemia	1 (3)	2 (8)	
AML with mutated *NPM1*	3 (10)	0 (0)	
AML with minimal differentiation	0 (0)	1 (4)	
AML without maturation	0 (0)	1 (4)	
AML with inv(3)(q21.3q26.2) or t(3;3)(q21.3;q26.2)	0 (0)	1 (4)	
AML with t(8;21)(q22;q22.1)	1 (3)	0 (0)	
AML with t(9;11)(p21.3;q23.3)	1 (3)	0 (0)	
Acute myelomonocytic leukemia	1 (3)	0 (0)	
**Allo-HSCT, n(%)**	6 (21)	3 (13)	0.487
**Cytogenetic risk category, n(%)**			**0.0107**
Favorable	1 (3)	0 (0)	
Intermediate	21 (72)	9 (38)	
Poor	8 (24)	15 (63)	
**ELN 2022 classification, n(%)**			**0.0095**
Favorable	3 (10)	0 (0)	
Intermediate	14 (48)	5 (21)	
Adverse	12 (41)	19 (79)	
**Ven_Aza risk classification, n(%)**			0.581
Higher benefit	17 (59)	10 (42)	
Intermediate	6 (21)	7 (29)	
Lower benefit	6 (21)	7 (29)	
**ELN 2024 Less-Intensive, n(%)**			0.466
Favorable	5 (17)	2 (8)	
Intermediate	19 (66)	15 (63)	
Adverse	5 (17)	7 (29)	
**Lab data at diagnosis**			
White blood cells (10^3^/ul)	3.8 (0.7-116)	3.8 (0.7-116)	0.74
Hemoglobin (g/dl)	8.45 (3.6-12.3)	8.4 (4.9-14.2)	0.981
Platelet (10^4^/ul)	5.5 (0.4-77.3)	7.65 (0.9-61.2)	0.484
Peripheral blood blast (%)	16 (0-94)	16 (0-98.5)	0.271
Bone marrow NCC (10^3^/ul)	96.1 (3.4-556)	61.6 (6.3-662)	0.768
Bone marrow blast (%)	32.6 (5.5-96.2)	42.2 (9.4-98.6)	0.397
CD7 positive/dim/negative/NA, n(%)	5(17)/3(10)/17(59)/4(14)	10(42)/0(0)/12(50)/2(8)	0.0666
CD56 positive/dim/negative/NA, n(%)	5(17)/4(14)/15(52)/5(17)	7(29)/2(8)/10(42)/5(21)	0.618
Peripheral blood *WT1* mRNA (copy/ugRNA)	14000 (50-200000)	33000 (64-270000)	0.462

Allo-HSCT; allogeneic hematopoietic stem cell transplantation, AML; acute myeloid leukemia, Aza; azacitidine, CR; complete remission, CRi; CR with incomplete hematologic recovery, ELN; European LeukemiaNet, NA; not available, NCC; nuclear cell count, Ven; venetoclax, *WT1*; Wilms tumor 1

### Type 1 and type 2 mutations and response to Aza/Ven

We next compared the genetic profiles of patients who achieved CR/CRi with those who did not. A total of 52 pathogenic variants were identified in the CR/CRi group and 38 in the non-CR group prior to Aza/Ven therapy. When treatment response was examined according to individual gene mutations, CR/CRi was more frequently observed in patients harboring *IDH1, IDH2, FLT3-TKD, PTPN11, NRAS, CBL, FLT3-ITD*, or *NPM1* mutations, whereas non-CR predominated among patients with *TP53, RUNX1*, or *KRAS* mutations (Fig 2C).

Based on the framework proposed by Makishima et al.[9], mutations were categorized into type 1 (*FLT3, PTPN11, WT1, IDH1/2, NPM1, NRAS*), type 2 (*GATA2, KRAS, TP53, RUNX1, STAG2, ASXL1, ZRSR2, TET2*), or other mutations. The distribution of mutation types differed significantly between the CR/CRi and non-CR groups, with type 1 mutations accounting for more than half of detected mutations in the CR/CRi group, whereas type 2 mutations predominated in the non-CR group (Fig 2D).

Baseline characteristics were compared between patients with Type 1 and Type 2 mutations (Table 3). Age, sex, WHO 2017 disease classification, and baseline laboratory parameters did not differ significantly between the two groups. In contrast, significant differences were observed in ELN 2022 classification, the Ven_Aza risk classification, and the ELN 2024 Less-Intensive classification. Patients with Type 2 mutations were more frequently classified into adverse-risk categories according to both the ELN 2022 and ELN 2024 Less-Intensive classifications, whereas patients with Type 1 mutations were more frequently classified into favorable or intermediate risk categories. In the Ven_Aza risk classification, Type 1 mutations were more frequently associated with higher benefit, whereas Type 2 mutations were more frequently associated with lower benefit.

**Table 3:** Patients’ characteristics.

Characteristics	Type 1 (n = 18)	Type 2 (n = 12)	p value
**Age, years, median**	71.5	74	0.524
< 65 years (%)	3 (17)	1 (8)	
65 −74 years (%)	8 (44)	5 (42)	
≥ 75 years (%)	7 (39)	6 (50)	
**Sex (M/F), n (%)**	9 (50)/9 (50)	7 (58)/5 (42)	0.722
**WHO 2017 disease classification, n (%)**			0.0231
AML with myelodysplasia-related changes	5 (28)	11 (92)	
AML with maturation	4 (22)	0 (0)	
AML with mutated *NPM1*	3 (17)	0 (0)	
Acute monoblastic/monocytic leukemia	1 (6)	1 (8)	
AML with inv(3)(q21.3q26.2) or t(3;3)(q21.3;q26.2)	1 (6)	0 (0)	
AML without maturation	1 (6)	0 (0)	
AML with minimal differentiation	1 (6)	0 (0)	
Acute myelomonocytic leukemia	1 (6)	0 (0)	
Therapy-related myeloid neoplasms	1 (6)	0 (0)	
**ELN 2022 classification, n(%)**			<0.001
Favorable	6 (33)	0 (0)	
Intermediate	10 (56)	0 (0)	
Adverse	2 (11)	12 (100)	
**Ven_Aza risk classification, n(%)**			0.0026
Higher benefit	9 (50)	2 (17)	
Intermediate	8 (44)	2 (17)	
Lower benefit	1 (6)	8 (67)	
**ELN 2024 Less intensity, n(%)**			<0.001
Favorable	7 (39)	0 (0)	
Intermediate	11 (61)	4 (33)	
Adverse	0 (0)	8 (67)	
**Gene mutations, n(%)**			<0.001
*NPM1*	10 (56)	0 (0)	
*FLT3-ITD*	5 (28)	0 (0)	
*NRAS*	4 (22)	0 (0)	
*IDH1*	3 (17)	0 (0)	
*IDH2*	3 (17)	0 (0)	
*PTPN11*	2 (11)	0 (0)	
*FLT3-TKD*	1 (6)	0 (0)	
*TP53*	0 (0)	8 (67)	
*KRAS*	0 (0)	3 (25)	
*GATA2*	0 (0)	2 (17)	
*RUNX1*	0 (0)	1 (8)	
**Lab data at diagnosis, median (range)**			
White blood cells (10^3^/ul)	16.3 (1.1-284.6)	3.6 (0.8-9.5)	0.133
Hemoglobin (g/dl)	8.6 (4.9-16.2)	8.3 (4.9-10.9)	0.985
Platelet (10^4^/ul)	7.5 (0.7-77.3)	6.5 (0.9-25.6)	0.52
Peripheral blood blast (%)	24.5 (0-98.5)	13.5 (0-54)	0.096
Bone marrow blast (%)	48.8 (5.5-98.6)	35.4 (9.4-79.2)	0.234
CD7 positive/dim/negative/NA, n(%)	4(22)/1(6)/9(50)/4(22)	5(42)/1(8)/6(50)/0(0)	0.832
CD56 positive/dim/negative/NA, n(%)	4(22)/0(0)/11(61)/3(17)	3(25)/1(8)/7(58)/1(8)	0.802
Peripheral blood *WT1* mRNA (copy/ugRNA)	46500 (64-200000)	29000 (50-270000)	0.608

AML; acute myeloid leukemia, ELN; European leukemiaNet, WT1; wilms tumor 1

Analysis of co-mutation patterns revealed that 34% of patients harbored Type 1 mutations alone, 23% harbored Type 2 mutations alone, 26% had neither Type 1 nor Type 2 mutations, and 17% had both mutation types (Fig 3A, 3B). Baseline characteristics were compared between patients with Type 1 mutations alone and those with Type 2 mutations alone (Table 3). Age, sex, and baseline laboratory parameters did not differ significantly between the two groups. In contrast, significant differences were observed in the WHO 2017 disease classification, ELN 2022 classification, Ven_Aza risk classification, and ELN 2024 Less-Intensive classification. Patients with Type 2 mutations alone were more frequently classified into adverse-risk categories according to both the ELN 2022 and ELN 2024 Less-Intensive classifications, whereas patients with Type 1 mutations alone were more frequently classified into favorable or intermediate-risk categories.

**Fig 3:**
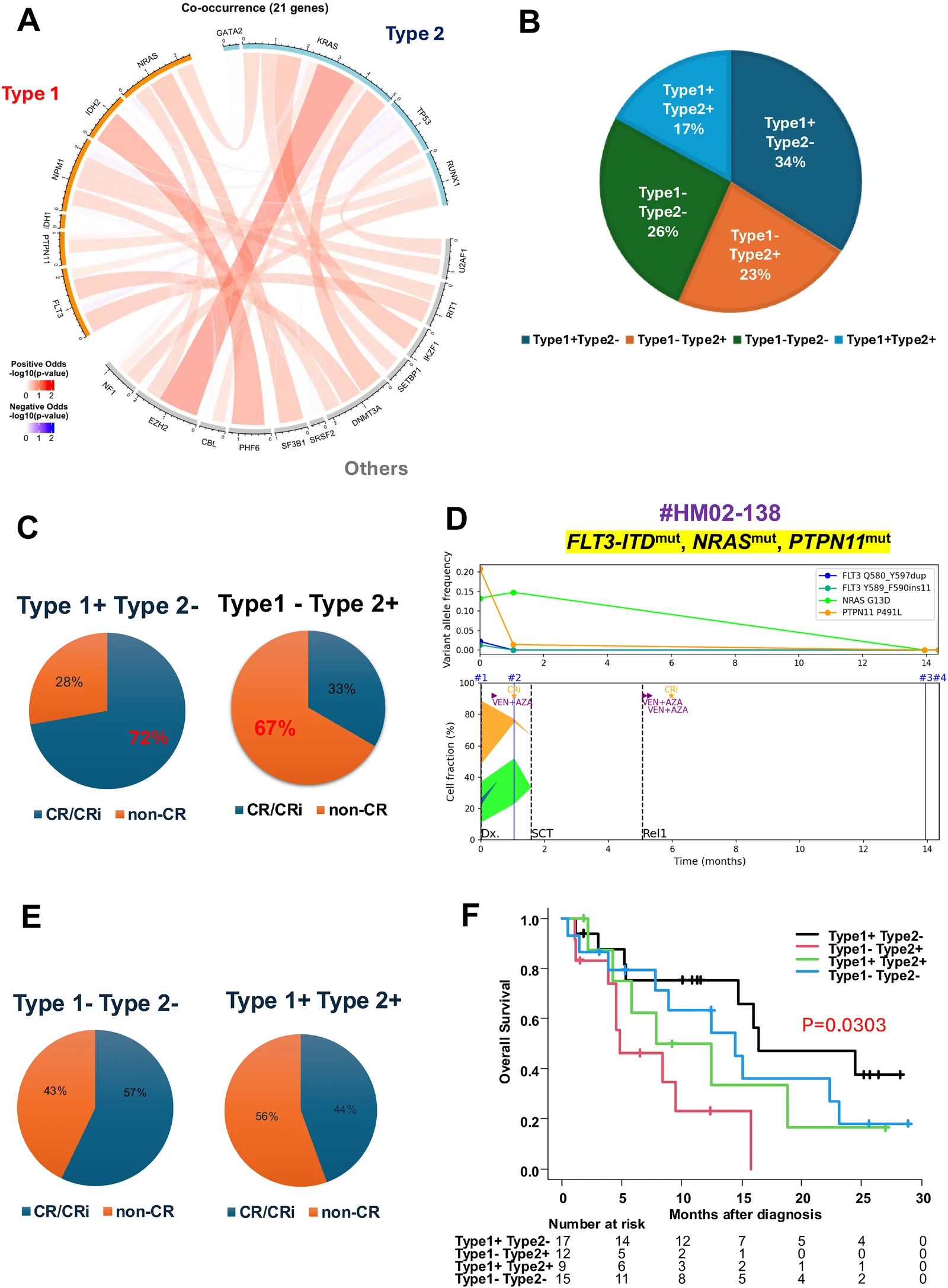
Clinical outcomes according to type 1 and type 2 mutations. A. Circos plot of 21 genetic mutations detected in this study. Genes are classified into three categories according to the outer arc colors: green indicates Type 1 mutations, purple indicates Type 2 mutations, and blue indicates other mutations. Ribbons connecting genes represent pairwise associations between mutations. Red ribbons indicate positive associations, whereas blue ribbons indicate negative associations; color intensity reflects the statistical significance of the association, expressed as −log10(P value). B. The distribution of gene mutations: type 1 mutations only, 34%; type 2 mutations only, 23%; neither type 1 nor type 2 mutations, 26%; and both types, 17%. C. Response rates according to gene groups: type 1+ and type 2- (CR/CRi 72%, non-CR 28%), type 1- and type 2+ (CR/CRi 33%, non-CR 67%). D. A representative case of type 1 mutation only (#HM02–138): An AML patient harbored *FLT3-ITD* (Q580_Y597dup, Y589_F590ins11), *NRAS* G13D, and *PTPN11* P491L mutations. The patient received Aza/Ven and achieved CRi, followed by allogeneic stem cell transplantation (allo-SCT). Although the patient relapsed after allo-SCT, two additional cycles of Aza/Ven induced a second remission. E. Response rates according to gene groups: type 1- and type 2- (CR/CRi 57%, non-CR 43%), type 1+ and type 2+ (CR/CRi 44%, non-CR 56%). F. Overall survival (OS) according to gene groups: type 1+ and type 2-; median OS 16.4 months (95% CI: 5.3-NA), type 1- and type 2 + ; median OS 4.9 months (95% CI: 1.2-NA), type 1+ and type 2 + ; median OS 10.2 months (95% CI: 2.2-NA), type 1- and type 2-; median OS 14.5 months (95% CI: 3.9–23.1) (p = 0.0303). OS differed significantly between the type 1 + /type 2 − group and the type 1 − /type 2 + group (p = 0.0023). Aza, azacitidine; CI, confidence interval; CR, complete remission; CRi, CR with incomplete hematologic recovery; Dx, diagnosis; OS, overall survival; NA, not available; Rel, relapse; SCT, stem cell transplantation; Ven, venetoclax.

CR/CRi was achieved in more than 70% of patients with Type 1 mutations alone, whereas approximately two-thirds of patients with Type 2 mutations alone were non-responders (Fig 3C). A similar trend was also observed among patients who received Aza/Ven as second-line or later therapy, although the number of evaluable patients was limited, with a CR rate of 75% in patients with Type 1 mutations alone and 0% in those with Type 2 mutations alone. Because AML-MRC accounted for 46% of the cohort, we additionally analyzed treatment responses stratified by AML-MRC status. A similar trend was observed in both AML-MRC and non–AML-MRC subgroups. Among patients with AML-MRC, the CR rate was 80% in patients with Type 1 mutations alone and 27% in those with Type 2 mutations alone. Among patients with non–AML-MRC, the CR rate was 69% in patients with Type 1 mutations alone; however, evaluation of Type 2 mutations in this subgroup was limited because only one patient had Type 2 mutations alone. A representative case demonstrated sustained sensitivity to Aza/Ven in the presence of type 1 mutations both before allogeneic transplantation and at post-transplant relapse (Fig 3D).

In contrast, among patients harboring both type 1 and type 2 mutations or neither mutation type, CR/CRi and non-CR occurred at similar frequencies (approximately 50% each), indicating limited discriminatory value of mutation status alone in these subgroups (Fig 3E). These mutation-defined subgroups were associated not only with remission rates but also with OS (Fig 3F). A significant difference in OS was observed among the four groups (p = 0.0303). In particular, patients in the Type 1-positive/Type 2-negative group had significantly longer OS than those in the Type 1-negative/Type 2-positive group (p = 0.0023).

### Mathematical model supports clinical decisions in Aza/Ven therapy

Although genetic mutation profiles were informative in approximately half of the patients, treatment response remained difficult to characterize in cases lacking dominant type 1 or type 2 mutations. To address this limitation, we developed a mathematical model integrating clinical variables to improve response prediction.

Given that response rates to Aza/Ven appeared comparable across treatment lines (Fig 1B), 17 Aza/Ven-treated cases lacking dominant type 1 or type 2 mutations were included in the modeling analysis irrespective of line of therapy. Using the same set of predictors, we compared LDA and SVM with class-imbalance weighting. Under LOOCV, the LDA model achieved an area under the ROC curve of 0.914 (95% CI, 0.744–1.000 by DeLong’s method) and an overall accuracy of 88.2% (Fig 4A). LDA was selected as the primary model based on overall accuracy and ROC–AUC.

**Fig 4:**
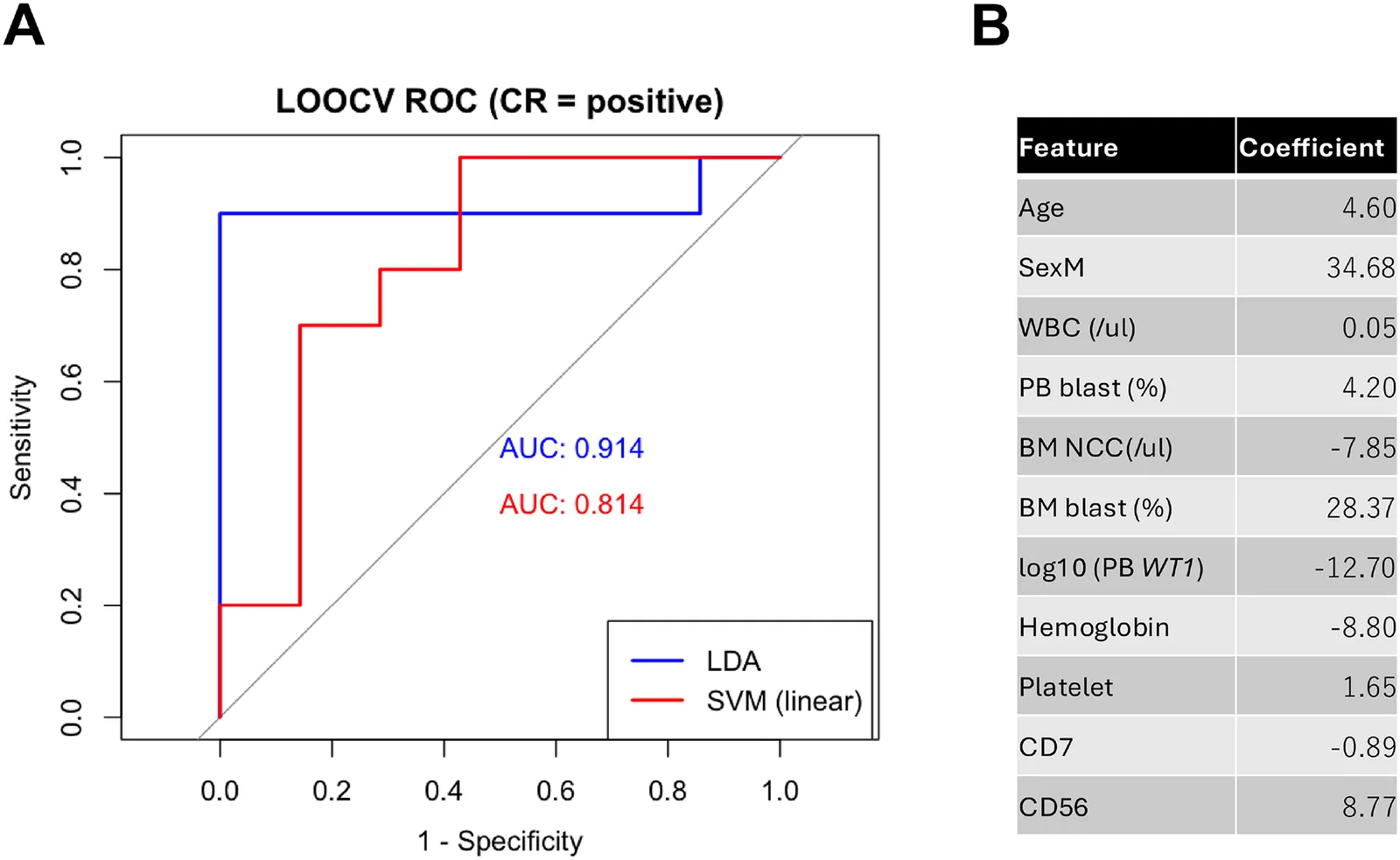
Feature coefficients in the multivariable prediction model. A. ROC curves and AUCs for two classification models (LDA and SVM) predicting response to azacitidine plus venetoclax (Aza/Ven) using baseline clinical factors in 17 evaluable cases lacking dominant type 1 or type 2 mutations. Model performance was evaluated by leave-one-out cross-validation (LOOCV). The LDA model achieved an AUC of 0.914 (95% CI, 0.744–1.000). B. Features and coefficients of the LDA model are shown. AUC, area under the curve; BM, bone marrow; CI, confidence interval; LDA, linear discriminant analysis; NCC, nuclear cell count; PB, peripheral blood; ROC, receiver operating characteristic; SVM, support vector machine; WBC, white blood cell; *WT1*, Wilms tumor 1.

To aid clinical interpretation, we examined the signs of the LDA coefficients shown in Fig 4B. Because CR/CRi was defined as the positive class, positive coefficients shifted the discriminant score toward predicted CR/CRi, whereas negative coefficients shifted it toward predicted non-CR. The model suggested that age, bone marrow blast percentage, peripheral blood blast percentage, CD56 expression, and the male-sex indicator were weighted toward CR/CRi prediction, whereas log10 peripheral blood WT1 expression, bone marrow nucleated cell count, and hemoglobin level were weighted toward non-CR prediction. These coefficients represent model-derived weights within the multivariable classifier and should not be interpreted as causal effects, particularly given the limited sample size and correlations among baseline variables.

In an exploratory cutoff-based analysis, the Youden index-derived cutoff for the LOOCV-predicted LDA probability was 0.0097. Using this cutoff, cases were classified into predicted responder and predicted non-responder groups. The predicted responder group showed a significantly higher CR/CRi rate than the predicted non-responder group (100.0% [9/9] vs 12.5% [1/8]; p = 0.000411). The cutoff-defined groups were highly concordant with the observed response categories, with 16 of 17 cases classified consistently with actual CR/CRi or non-CR status.

OS was also assessed using the same cutoff. The predicted responder group showed a numerically higher 12-month OS probability than the predicted non-responder group (77.8% vs 60.0%); however, the difference was not statistically significant (p = 0.445). Because the cutoff-defined groups were almost identical to the observed response categories and the number of evaluable cases was limited, this OS analysis was not interpreted as independent prognostic validation of the model.

## Discussion

In this prospective observational study, we evaluated the real-world activity of Aza/Ven, focusing on the relationship between the genetic background of AML and treatment response using mutation clusters described in a prior report [9]. Overall, Aza/Ven demonstrated comparable clinical activity across different treatment lines. Notably, in the first-line setting, patients harboring type 1 mutations alone were more likely to achieve CR/CRi, whereas those with type 2 mutations alone showed lower response rates. In addition, a mathematical model integrating clinical variables helped characterize treatment responsiveness even in cases without identifiable type 1 or type 2 mutations.

The classification of type 1 and type 2 mutations was originally proposed to describe clonal architecture and evolutionary timing in secondary AML (sAML) and high-risk MDS, rather than to predict treatment response [9]. Specifically, type 1 mutations are late-acquired alterations emerging during progression from high-risk MDS to sAML, whereas type 2 mutations are enriched in earlier disease stages and high-risk MDS. In the present study, more than half of the patients treated with first-line Aza/Ven had AML-MRC with an antecedent MDS background (Table 2). This clinical distribution likely reflects the historical establishment of azacitidine as a standard therapy for high-risk MDS [18], leading clinicians to favor azacitidine-containing regimens, including Aza/Ven, in this population.

A previous study has reported that AML cases harboring type 1 mutations alone have an inferior prognosis compared with those carrying only type 2 mutations [9]. In contrast, our findings demonstrated a different pattern, in which patients with type 1 mutations exhibited higher response rates to Aza/Ven and superior overall survival compared with those harboring type 2 mutations. These results do not suggest that type 1 mutations are inherently favorable prognostic markers but rather indicate that Aza/Ven may preferentially induce remission in AML characterized by type 1–dominant clonal architecture.

Several studies have investigated the relationship between genetic alterations and response to Aza/Ven therapy in AML. In the pivotal clinical trial, first-line Aza/Ven demonstrated high efficacy in patients with *IDH* mutations [20], a finding that was recapitulated in our cohort. Subsequent analyses have suggested that the benefit of Aza/Ven may be stratified by the presence or absence of mutations such as *TP53, FLT3-ITD, KRAS*, and *NRAS*, with higher benefit observed in patients lacking these alterations [17]. Other reports have similarly identified *TP53* and *RAS* pathway mutations as adverse factors for Aza/Ven outcomes [19]. The ELN 2024 Less-Intensive classification, which incorporates these findings, has been reported [16]. Although the present cohort could not be fully classified according to this system because ivosidenib plus azacitidine was not used in *IDH1*-mutated cases and *DDX41* was not included in our genomic panel testing, provisional application of the ELN 2024 Less-Intensive criteria demonstrated a significant difference in OS. Conversely, CR/CRi rates showed a decreasing trend with increasing risk category — favorable, 71.4%; intermediate, 54.3%; and adverse, 41.7% — but the difference was not statistically significant.

Consistent with prior literature, *TP53*-mutated AML in our study showed poor responses to Aza/Ven.

In contrast to *TP53*, the impact of *RAS* mutations on treatment response remains heterogeneous across studies. While some reports have shown that both *KRAS* and *NRAS* mutations are associated with inferior outcomes following hypomethylating agent–based therapy [17,19], other studies have suggested that *KRAS* mutations confer resistance whereas *NRAS* mutations do not uniformly impair treatment response [20,21]. In our cohort, *KRAS* mutations were associated with a higher proportion of non-responders, whereas *NRAS* mutations did not appear to adversely affect Aza/Ven response. These discrepancies may reflect differences in co-mutation patterns, specific mutational loci, or population-specific genetic backgrounds, including potential racial differences.

Recent molecular studies provide a biological framework that may explain these observations. Shimony et al. reported that Aza/Ven therapy is particularly effective in sAML [22]. Similarly, Winters et al. demonstrated through clonal tracking analyses that late driver mutations, particularly signaling-related alterations, tend to regress under Aza/Ven therapy, whereas MDS-related or pre-leukemic mutations frequently persist [10]. These patterns reflect hierarchical clonal dependency. Type 1 mutations represent late-acquired proliferative subclones that arise during leukemic transformation and may exhibit heightened dependence on *BCL-2*–mediated oxidative phosphorylation. Alterations such as *NPM1* or *IDH* mutations, in particular, appear to confer vulnerability to Aza/Ven through synergistic effects of hypomethylating agent–induced cellular stress and BCL-2 inhibition [23]. By contrast, although the type 2 mutation group comprises genetically heterogeneous alterations, these genes share a common biological feature in that they are typically acquired early during disease evolution and primarily affect hematopoietic stem cell function, differentiation, or genomic stability, rather than directly driving leukemic proliferation. Consequently, persistence of type 2 mutations after azacitidine plus venetoclax likely reflects resistance at the level of underlying disease architecture, rather than insufficient suppression of late leukemic clones [10,22].

Mathematical modeling offers a complementary approach to mutation-based stratification by integrating multidimensional clinical variables to estimate treatment responsiveness. In this study, exploratory modeling demonstrated reasonable discriminatory performance, including in patients lacking established prognostic mutations. While such models are not intended to replace molecular diagnostics, they may support the interpretation of response heterogeneity in genetically uninformative AML [24].

Another noteworthy observation was that response rates to Aza/Ven appeared broadly comparable between first-line and later-line settings. Unlike conventional cytotoxic chemotherapy, for which response rates typically decline with successive lines of therapy due to clonal evolution and resistance, Aza/Ven may retain activity in selected relapsed or refractory cases. However, given the observational design and limited sample size, these findings should be interpreted cautiously, and further studies are required to define the patient subsets most likely to benefit from later-line Aza/Ven therapy.

This study has several limitations. First, the sample size was relatively small, which limited the statistical power for gene-specific and subgroup analyses. Accordingly, all findings, including those derived from predictive modeling, should be considered exploratory and require validation in larger, independent cohorts. In particular, whether the Type 1/Type 2 mutation classification provides predictive or prognostic information independent of established prognostic factors, including ELN risk classifications, remains unclear. Because of the limited sample size and the small number of patients in each mutation-defined subgroup, a robust multivariable model adjusting for established prognostic factors could not be performed. Moreover, the observed associations between Type 1/Type 2 mutations and treatment outcomes may be partly influenced by established genetic risk classifications, as Type 1 genes generally correspond to favorable-to-intermediate risk categories, whereas Type 2 genes are more frequently associated with intermediate-to-adverse risk categories in the ELN 2022/2024 classifications.

Second, although the LDA/SVM model with LOOCV showed high performance, including an AUC of 0.914, no external validation cohort was available; therefore, the possibility of overfitting cannot be excluded. Obtaining an independent cohort with comparable genomic and clinical data in patients treated with Aza/Ven remains challenging, but external validation is essential before this model can be applied in clinical practice. Thus, the present model should be regarded as exploratory and hypothesis-generating rather than clinically implementable at this stage.

Third, detailed clinical information, including performance status, comorbidities, and treatment dosing, was not available for all patients, and adverse events and treatment tolerability were not systematically evaluated. Therefore, the potential impact of these clinical and safety-related factors could not be fully assessed. Finally, prognostic interpretation was challenging in patients harboring both Type 1 and Type 2 mutations. Future studies incorporating variant allele frequency, co-mutation architecture, longitudinal clonal dynamics, and both efficacy and toxicity data may further refine prediction of Aza/Ven response and better define the clinical implications of these mutation-defined subgroups.

In conclusion, Aza/Ven demonstrated consistent clinical activity across treatment lines in this cohort, while response patterns differed according to underlying mutational architecture. The integration of clonal frameworks with mathematical modeling may provide biologically interpretable insights into heterogeneous treatment responses, warranting further validation in larger studies.

## Supporting information

S1 FileEstimation of LDA coefficients using the entire dataset.The preprocessed data were converted to a design matrix using dummy coding for all factors, with the response Y coded as 0 for nonCR and 1 for CR. Let X denote the resulting predictor matrix, and let $\mu_{\text{1}}$ and $\mu_{\text{2}}$ be the mean vectors of the predictors in the nonCR and CR groups, respectively. The within-class covariance matrices $S_{\text{1}}$ and $S_{\text{2}}$ were computed for each group, and a pooled within-class covariance matrix, $\Sigma_{p}=\frac{(n_{\text{1}}-\text{1})S_{\text{1}}+(n_{\text{2}}-\text{1})S_{\text{2}}}{n_{\text{1}}+n_{\text{2}}-\text{2}}$, was then formed, where $n_{\text{1}}$and $n_{\text{2}}$are the numbers of nonCR and CR cases. The discriminant weight vector β was obtained as $\beta =\Sigma_{p}^{-\text{1}}(\mu_{\text{2}}-\mu_{\text{1}}),$ and prior class probabilities $\pi_{\text{1}}$ and $\pi_{\text{2}}$ were set equal to the empirical class proportions. The scalar intercept $\beta_{\text{0}}$ for the two-group discriminant function was calculated as $\beta_{\text{0}}=-\frac{\text{1}}{\text{2}}(\mu_{\text{2}}^{⊤}\Sigma_{p}^{-\text{1}}\mu_{\text{2}}-\mu_{\text{1}}^{⊤}\Sigma_{p}^{-\text{1}}\mu_{\text{1}})+\text{log}(\frac{\pi_{\text{2}}}{\pi_{\text{1}}}).\;$For each subject with predictor vector x, the linear discriminant score was defined as $\text{score}(x)=\beta_{\text{0}}+\beta^{⊤}x,$ and classification was given by score(x)>0 for CR and score(x)≤0 for nonCR. The elements of β thus quantify the contribution of each standardized predictor to shifting a patient towards the CR or nonCR side of the discriminant boundary.(DOCX)
