# Supplementary material for: Genomic and clinical determinants of response to Azacitidine plus venetoclax in acute myeloid leukemia: Results from the HM-SCREEN-Japan 02 study
Source: PLoS One. 2026 Oct 1;21(10):e0357898. doi: 10.1371/journal.pone.0357898 (PMC13630213; doi:10.1371/journal.pone.0357898)
Supplement: Supplementary file 1 [file pone.0357898.s001.docx]

**Supplemental materials**

**Estimation of LDA coefficients using the entire dataset.**

The preprocessed data were converted to a design matrix using dummy coding for all factors, with the response $Y$ coded as 0 for nonCR and 1 for CR. Let $X$ denote the resulting predictor matrix, and let $\mu_{1}$ and $\mu_{2}$ be the mean vectors of the predictors in the nonCR and CR groups, respectively. The within-class covariance matrices $S_{1}$ and $S_{2}$ were computed for each group, and a pooled within-class covariance matrix, $\Sigma_{p}=\frac{(n_{1}-1)S_{1}+(n_{2}-1)S_{2}}{n_{1}+n_{2}-2}$, was then formed, where $n_{1}$and $n_{2}$are the numbers of nonCR and CR cases. The discriminant weight vector $\beta$ was obtained as $\beta=\Sigma_{p}^{-1}(\mu_{2}-\mu_{1}),$ and prior class probabilities $\pi_{1}$ and $\pi_{2}$ were set equal to the empirical class proportions. The scalar intercept $\beta_{0}$ for the two-group discriminant function was calculated as $\beta_{0}=-\frac{1}{2}(\mu_{2}^{\top}\Sigma_{p}^{-1}\mu_{2}-\mu_{1}^{\top}\Sigma_{p}^{-1}\mu_{1})+\log(\frac{\pi_{2}}{\pi_{1}}).$For each subject with predictor vector $x$, the linear discriminant score was defined as $\text{score}(x)=\beta_{0}+\beta^{\top}x,$ and classification was given by $\text{score}(x)>0$ for CR and $\text{score}(x)\leq0$ for nonCR. The elements of $\beta$ thus quantify the contribution of each standardized predictor to shifting a patient towards the CR or nonCR side of the discriminant boundary.
