# Supplementary material for: Genomic and clinical determinants of response to Azacitidine plus venetoclax in acute myeloid leukemia: Results from the HM-SCREEN-Japan 02 study
Source: PLoS One. 2026 Oct 1;21(10):e0357898. doi: 10.1371/journal.pone.0357898 (PMC13630213; doi:10.1371/journal.pone.0357898)
Supplement: Supplementary file 3 [file pone.0357898.s004.docx]

**Reviewer #1:**

**The authors have satisfactorily addressed the majority of the reviewers' concerns through additional analyses, clarification of the methodology, and a more balanced discussion of the limitations. Only minor revisions are still warranted, mainly to further temper the interpretation of exploratory subgroup analyses and to clarify that the predictive model is not yet ready for clinical implementation without external validation. Although several additional subgroup analyses have been performed, it remains unclear whether the Type 1/Type 2 mutation classification provides independent predictive information beyond established prognostic factors. If a multivariable model cannot be performed because of the limited sample size, this limitation should be explicitly acknowledged. These remaining issues do not substantially affect the overall quality of the work.**

**Response:**

Thank you for your thoughtful and constructive comments. We appreciate the reviewer’s positive assessment of the revised manuscript. We agree that the interpretation of the exploratory subgroup analyses should be further tempered and that the predictive model should not be considered ready for clinical implementation without external validation.

In response to this comment, we revised the Discussion to explicitly acknowledge that whether the Type 1/Type 2 mutation classification provides predictive information independent of established prognostic factors, including ELN risk classifications, remains unclear. Because of the limited sample size and the small number of patients in each mutation-defined subgroup, a robust multivariable model adjusting for established prognostic factors could not be performed. We also clarified that the LDA/SVM model should be regarded as exploratory and hypothesis-generating, and that external validation in larger independent cohorts is essential before clinical implementation (pages 35-36, lines 524-541).

**Reviewer #2:**

**The authors have adequately addressed the reviewer’s comments and provided appropriate revisions throughout the manuscript. The responses are clear and comprehensive, and the revisions have improved the overall quality and clarity of the manuscript. I have no further major concerns regarding this submission.**

**Response:**

We sincerely thank the reviewer for the positive evaluation of our revised manuscript. We are pleased that the revisions have addressed the reviewer’s concerns and improved the clarity and quality of the manuscript.
