# Supplementary material for: Genomic and clinical determinants of response to Azacitidine plus venetoclax in acute myeloid leukemia: Results from the HM-SCREEN-Japan 02 study
Source: PLoS One. 2026 Oct 1;21(10):e0357898. doi: 10.1371/journal.pone.0357898 (PMC13630213; doi:10.1371/journal.pone.0357898)
Supplement: Peer review history

# Peer review: Decision Letter 0

**DOI:** 10.1371/journal.pone.0357898.r001

**Authors:** Francesco Bertolini

27 Apr 2026

Dear Dr. Minami,

Thank you for submitting your manuscript to PLOS ONE. After careful consideration, we feel that it has merit but does not fully meet PLOS ONE’s publication criteria as it currently stands. Therefore, we invite you to submit a revised version of the manuscript that addresses the points raised during the review process by both Reviewers, experts in the field.

Please submit your revised manuscript by Jun 11 2026 11:59PM. If you will need more time than this to complete your revisions, please reply to this message or contact the journal office at plosone@plos.org. When you're ready to submit your revision, log on to https://www.editorialmanager.com/pone/ and select the 'Submissions Needing Revision' folder to locate your manuscript file.

- A letter that responds to each point raised by the academic editor and reviewer(s). You should upload this letter as a separate file labeled 'Response to Reviewers'.
- A marked-up copy of your manuscript that highlights changes made to the original version. You should upload this as a separate file labeled 'Revised Manuscript with Track Changes'.
- An unmarked version of your revised paper without tracked changes. You should upload this as a separate file labeled 'Manuscript'.

If you would like to make changes to your financial disclosure, please include your updated statement in your cover letter. Guidelines for resubmitting your figure files are available below the reviewer comments at the end of this letter.

If applicable, we recommend that you deposit your laboratory protocols in protocols.io to enhance the reproducibility of your results. Protocols.io assigns your protocol its own identifier (DOI) so that it can be cited independently in the future. For instructions see: https://journals.plos.org/plosone/s/submission-guidelines#loc-laboratory-protocols. Additionally, PLOS ONE offers an option for publishing peer-reviewed Lab Protocol articles, which describe protocols hosted on protocols.io. Read more information on sharing protocols at https://plos.org/protocols?utm_medium=editorial-email&utm_source=authorletters&utm_campaign=protocols.

As the corresponding author, your ORCID iD is verified in the submission system and will appear in the published article. PLOS supports the use of ORCID, and we encourage all coauthors to register for an ORCID iD and use it as well. Please encourage your coauthors to verify their ORCID iD within the submission system before final acceptance, as unverified ORCID iDs will not appear in the published article. *Only* the individual author can complete the verification step; PLOS staff *cannot* verify ORCID iDs on behalf of authors.

We look forward to receiving your revised manuscript.

Kind regards,

Francesco Bertolini, MD, PhD

Academic Editor

PLOS One

**Journal Requirements:**

When submitting your revision, we need you to address these additional requirements.

1. Please ensure that your manuscript meets PLOS ONE's style requirements, including those for file naming. The PLOS ONE style templates can be found at

https://journals.plos.org/plosone/s/file?id=wjVg/PLOSOne_formatting_sample_main_body.pdfand

https://journals.plos.org/plosone/s/file?id=ba62/PLOSOne_formatting_sample_title_authors_affiliations.pdf

2. We note that the grant information you provided in the ‘Funding Information’ and ‘Financial Disclosure’ sections do not match.

When you resubmit, please ensure that you provide the correct grant numbers for the awards you received for your study in the ‘Funding Information’ section.

3. Thank you for stating the following financial disclosure:

“Grant (2021-A-11), funded by the National Cancer Center, Japan.”

Please state what role the funders took in the study.  If the funders had no role, please state: "The funders had no role in study design, data collection and analysis, decision to publish, or preparation of the manuscript."

If this statement is not correct you must amend it as needed.

Please include this amended Role of Funder statement in your cover letter; we will change the online submission form on your behalf.

4. Thank you for stating the following in the Competing Interests section:

“Tomoaki Ueda: Chugai (Honoraria). Kentaro Fukushima: Janssen Pharma, Pfizer Japan Inc. (Honoraria). Goichi Yoshimoto: AbbVie (Honoraria). Motoshi Ichikawa: Otsuka Pharma KK, Incyte Japan. Naoto Takahashi: : Otsuka Pharma KK, Incyte Japan, Otsuka Pharma KK, Astellas Pharma Inc. (Research Funding), Novartis Pharma KK, Otsuka Pharma KK (Honoraria). Naoko Hosono: Abbie, Astellas, Nippon Shinyaku (Honoraria). Takahiro Yamauchi: Abbie, Astellas, Nihon-Shinyaku (Research Funding), Nihon-Shinyaku, Abbvie (Honoraria). Takeshi Kondo: Yahoo Japan Foundation (Research Funding), Pfizer Japan Inc., Otsuka Pharmaceutical, Novartis Pharma KK, Bristol-Myers Squibb (Honoraria). Shigeru Kusumoto: BMS, Meiji Seika Pharma, AbbVie (Research Funding), Janssen, Daiichi Sankyo, Ono, Chugai, Takeda, Sanofi, AbbVie, Meiji Seika Pharma, Eisai, Mundi Pharma, Kyowa Kirin, Genmab, AstraZeneca, Nippon Shiyaku (Honoraria). Junya Kuroda: Bristol-Myers Squibb, Sanofi, Janssen Pharmaceutical, Pfizer, Ono Pharmaceutical, Chugai Pharmaceutical, Novartis (Honoraria). Yosuke Minami: Bristol-Myers Squibb Company, Novartis Pharma KK, Pfizer Japan Inc., Takeda (Honoraria).”

We note that you received funding from a commercial sources:

- Janssen Pharma, Pfizer Japan Inc
- AbbVie
- Otsuka Pharma KK,
- Incyte Japan
- Astellas Pharma Inc.
- Novartis Pharma KK
- Nippon Shinyaku
- Pfizer Japan Inc.
- Otsuka Pharmaceutical
- Bristol-Myers Squibb
- Meiji Seika Pharma
- Eisai, Mundi Pharma
- Kyowa Kirin
- Genmab
- AstraZeneca
- Ono Pharmaceutical
- Chugai Pharmaceutical

Please provide an amended Competing Interests Statement that explicitly states this commercial funder, along with any other relevant declarations relating to employment, consultancy, patents, products in development, marketed products, etc.

Within this Competing Interests Statement, please confirm that this does not alter your adherence to all PLOS ONE policies on sharing data and materials by including the following statement: "This does not alter our adherence to PLOS ONE policies on sharing data and materials.” (as detailed online in our guide for authors http://journals.plos.org/plosone/s/competing-interests).  If there are restrictions on sharing of data and/or materials, please state these. Please note that we cannot proceed with consideration of your article until this information has been declared.

Please include your amended Competing Interests Statement within your cover letter. We will change the online submission form on your behalf.

5. Please note that funding information should not appear in any section or other areas of your manuscript. We will only publish funding information present in the Funding Statement section of the online submission form. Please remove any funding-related text from the manuscript.

6. In the online submission form you indicate that your data is not available for proprietary reasons and have provided a contact point for accessing this data. Please note that your current contact point is a co-author on this manuscript. According to our Data Policy, the contact point must not be an author on the manuscript and must be an institutional contact, ideally not an individual. Please revise your data statement to a non-author institutional point of contact, such as a data access or ethics committee, and send this to us via return email. Please also include contact information for the third party organization, and please include the full citation of where the data can be found.

7. Your ethics statement should only appear in the Methods section of your manuscript. If your ethics statement is written in any section besides the Methods, please move it to the Methods section and delete it from any other section. Please ensure that your ethics statement is included in your manuscript, as the ethics statement entered into the online submission form will not be published alongside your manuscript.

8. Please include captions for your Supporting Information files at the end of your manuscript, and update any in-text citations to match accordingly. Please see our Supporting Information guidelines for more information: http://journals.plos.org/plosone/s/supporting-information.

9. If the reviewer comments include a recommendation to cite specific previously published works, please review and evaluate these publications to determine whether they are relevant and should be cited. There is no requirement to cite these works unless the editor has indicated otherwise.

[Note: HTML markup is below. Please do not edit.]

Reviewers' comments:

Reviewer's Responses to Questions

**Comments to the Author**

1. Is the manuscript technically sound, and do the data support the conclusions?

Reviewer #1: Partly

Reviewer #2: Yes

**********

2. Has the statistical analysis been performed appropriately and rigorously?

Reviewer #1: Yes

Reviewer #2: Yes

**********

3. Have the authors made all data underlying the findings in their manuscript fully available?

The PLOS Data policy

Reviewer #1: Yes

Reviewer #2: Yes

**********

4. Is the manuscript presented in an intelligible fashion and written in standard English?

Reviewer #1: Yes

Reviewer #2: Yes

**********

Reviewer #1: This manuscript presents a prospective multicenter observational study evaluating genomic and clinical predictors of response to azacitidine plus venetoclax (Aza/Ven) in patients with acute myeloid leukemia (AML). The authors integrate targeted next-generation sequencing, cytogenetics, and clinical variables, and further develop a predictive model for treatment response.

The study addresses a clinically important question, as response heterogeneity to Aza/Ven remains a major challenge in AML management. The application of a type 1/type 2 mutation framework and integration of statistical modeling are notable strengths. However, several methodological and interpretative limitations restrict the robustness and generalizability of the conclusions.

MAJOR POINTS

- Describe patients according to WHO-2022 classification

- Association between first-line Aza/Ven response and overall survival should be done according to ELN-24 risk groups.

- Detail median and range of total AZA-Ven cycles administered.

- The predictive modeling (LDA/SVM with LOOCV) demonstrates high performance (AUC 0.914), but there is no external validation cohort. Given the small dataset and use of LOOCV, the model is at risk of overfitting. Validation in an independent cohort is essential before clinical applicability and should be highly desirable.

- Patients across first-line, second-line, and later treatment settings are combined in several analyses. Although the authors justify this approach, the biological and clinical heterogeneity may confound interpretation of results, particularly for modeling analyses.´

MINOR POINTS

1. Some figures (e.g., mutation distribution and circos plots) are difficult to interpret without more detailed legends.

2. Consider simplifying or annotating key findings more clearly.

3. The manuscript alternates between “CR/CRi” and “CR/CR.” Terminology should be standardized throughout.

4. While the modeling methodology is well described, the clinical interpretability of the model coefficients could be further emphasized in the main text rather than supplementary materials.

5. Important clinical factors such as performance status, comorbidities, and treatment dosing are not included. The absence of these variables should be acknowledged.

6. The manuscript focuses on efficacy but does not address adverse events or tolerability, which are important for clinical interpretation.

Reviewer #2: In this study, the authors analyzed patients with AML treated with azacitidine/venetoclax (Aza/Ven) in the prospective HM-SCREEN-Japan 02 study using targeted NGS of 53 genes, cytogenetic analyses, and clinical variables. They demonstrated that mutations classified as type 1 and type 2 were associated with CR rates in patients with AML receiving first-line Aza/Ven therapy. Although the study presents interesting findings, several issues should be addressed.

________________________________________

Major Comments

1. This cohort includes patients with AML treated with Aza/Ven, among whom 46% were classified as AML-MRC. This distribution appears to be skewed compared with that of general AML populations. As both type 1 and type 2 mutations examined in this study are frequently associated with MDS-related AML, the results may be influenced by this cohort composition. Indeed, conflicting effects of type 1 and type 2 mutations on AML treatment outcomes have been reported. The authors should consider analyzing the impact of type 1 and type 2 mutations separately in AML-MRC and non–AML-MRC subgroups.

2. Differences in baseline characteristics and cytogenetic findings between patients with type 1 and type 2 mutations should be clarified. In addition, the frequencies of individual gene mutations should be presented for both groups. The probability of overall survival (OS) should also be compared between the type 1 and type 2 mutation groups.

3. Type 1 and type 2 genes generally correspond to favorable-to-intermediate and intermediate-to-adverse risk categories, respectively, in the ELN 2022/2024 classification. This potential confounding effect should be discussed.

4. According to the NGS data, 11 patients initially classified as intermediate risk based on cytogenetics alone were reclassified as favorable or adverse risk according to the ELN 2022 risk stratification. The specific genetic abnormalities responsible for this reclassification should be described.

5. Recently, the ELN 2024 genetic risk classification for patients with newly diagnosed AML receiving less intensive HMA-based therapies (ELN 2024 Less-Intensive) has been proposed. The authors are encouraged to reclassify the study cohort according to this system. Furthermore, patient characteristics (e.g., Table 2), stratified by type 1 and type 2 mutations, should be compared with the ELN 2024 Less-Intensive classification.

6. The CR rates were reported to be similar across first-line, second-line, and third-line or later Aza/Ven therapy. The number of treatment cycles required to achieve CR in each setting should be provided.

7. The association between type 1 and type 2 mutations and outcomes following second-line or later Aza/Ven therapy should also be evaluated.

8. Although the authors propose a mathematical model integrating baseline clinical variables, even in patients without established prognostic gene mutations, its added value in the context of NGS-based analyses remains unclear. The number of patients included in this model and the 95% confidence interval of the area under the ROC curve should be provided. Furthermore, using the ROC-derived cutoff value, CR rates and OS probabilities could be assessed.

________________________________________

Minor Comments

1. In the legend of Figure 1c, is “CRR by Aza/Ven line of therapy (Chemotherapy)” correct?

2. References should be provided for the statements in lines 269–270 and 370–371.

3. The definitions of negative, dim, and positive expression for CD7 and CD56 should be clearly described.

**********

what does this mean?). If published, this will include your full peer review and any attached files.

If you choose “no”, your identity will remain anonymous but your review may still be made public.

**Do you want your identity to be public for this peer review?** For information about this choice, including consent withdrawal, please see our Privacy Policy

Reviewer #1: No

Reviewer #2: No

**********

[NOTE: If reviewer comments were submitted as an attachment file, they will be attached to this email and accessible via the submission site. Please log into your account, locate the manuscript record, and check for the action link "View Attachments". If this link does not appear, there are no attachment files.]

To ensure your figures meet our technical requirements, please review our figure guidelines: https://journals.plos.org/plosone/s/figures

You may also use PLOS’s free figure tool, NAAS, to help you prepare publication quality figures: https://journals.plos.org/plosone/s/figures#loc-tools-for-figure-preparation.

NAAS will assess whether your figures meet our technical requirements by comparing each figure against our figure specifications.

# Author comment: Author response to Decision Letter 1

**DOI:** 10.1371/journal.pone.0357898.r002

3 Jun 2026

Please ensure that your manuscript meets PLOS ONE's style requirements, including those for file naming.

Response: Thank you for this instruction. We have revised the manuscript to comply with PLOS ONE’s style requirements. We have also checked the file names for the manuscript, figures, and Supporting Information files and revised them to match the journal’s file naming requirements.

We note that the grant information you provided in the ‘Funding Information’ and ‘Financial Disclosure’ sections do not match. When you resubmit, please ensure that you provide the correct grant numbers for the awards you received for your study in the ‘Funding Information’ section.

Response: Thank you for pointing this out. We have carefully checked the grant information in both the Funding Information and Financial Disclosure sections and corrected the grant numbers so that they are consistent. The Funding Information section now includes the correct grant numbers for the awards that supported this study.

Thank you for stating the following financial disclosure: “Grant (2021-A-11), funded by the National Cancer Center, Japan.” Please state what role the funders took in the study. If the funders had no role, please state: "The funders had no role in study design, data collection and analysis, decision to publish, or preparation of the manuscript." If this statement is not correct you must amend it as needed. Please include this amended Role of Funder statement in your cover letter; we will change the online submission form on your behalf.

Response: Thank you for this instruction. We have included the amended Role of Funder statement in the cover letter, as requested. The statement reads: “Grant 2021-A-11 was funded by the National Cancer Center, Japan. The funders had no role in study design, data collection and analysis, decision to publish, or preparation of the manuscript.”

Please provide an amended Competing Interests Statement that explicitly states this commercial funder, along with any other relevant declarations relating to employment, consultancy, patents, products in development, marketed products, etc. Within this Competing Interests Statement, please confirm that this does not alter your adherence to all PLOS ONE policies on sharing data and materials.

Response: Thank you for this instruction. We have amended the Competing Interests Statement to explicitly state all commercial sources of honoraria and research funding. We have also included a statement confirming that the declared competing interests do not alter our adherence to PLOS ONE policies on sharing data and materials. The amended Competing Interests Statement has been included in the cover letter as requested.

Please note that funding information should not appear in any section or other areas of your manuscript. We will only publish funding information present in the Funding Statement section of the online submission form. Please remove any funding-related text from the manuscript.

Response: Thank you for this instruction. We have removed all funding-related text from the manuscript. Funding information is provided only in the Funding Statement section of the online submission form, in accordance with PLOS ONE requirements.

Your ethics statement should only appear in the Methods section of your manuscript. If your ethics statement is written in any section besides the Methods, please move it to the Methods section and delete it from any other section. Please ensure that your ethics statement is included in your manuscript, as the ethics statement entered into the online submission form will not be published alongside your manuscript.

Response: Thank you for this instruction. We have ensured that the ethics statement appears only in the Methods section of the manuscript. Any ethics-related text outside the Methods section has been removed. The ethics statement remains included in the manuscript because the ethics statement entered into the online submission form will not be published alongside the manuscript.

Please include captions for your Supporting Information files at the end of your manuscript, and update any in-text citations to match accordingly.

Response: Thank you for this instruction. We included all the Supporting Information at the end of the manuscript.

If the reviewer comments include a recommendation to cite specific previously published works, please review and evaluate these publications to determine whether they are relevant and should be cited. There is no requirement to cite these works unless the editor has indicated otherwise.

Response: Thank you for this guidance. The reviewer comments did not include any recommendation to cite specific previously published works; therefore, no additional citations were added in response to this item.

Reviewer #1: This manuscript presents a prospective multicenter observational study evaluating genomic and clinical predictors of response to azacitidine plus venetoclax (Aza/Ven) in patients with acute myeloid leukemia (AML). The authors integrate targeted next-generation sequencing, cytogenetics, and clinical variables, and further develop a predictive model for treatment response.

The study addresses a clinically important question, as response heterogeneity to Aza/Ven remains a major challenge in AML management. The application of a type 1/type 2 mutation framework and integration of statistical modeling are notable strengths. However, several methodological and interpretative limitations restrict the robustness and generalizability of the conclusions.

Response: We sincerely thank the reviewer for the thorough review and accurate analysis of our manuscript. We are also thankful for the positive comments and constructive suggestions for improving our work. We have modified our manuscript and figures as follows.

Describe patients according to WHO-2022 classification.

Response: Thank you for this helpful suggestion. In response to the reviewer’s comment, we added the disease classification according to the WHO 2022 classification to Table 1 (page 13) in the revised manuscript. Although the study protocol specified the use of the WHO 2017 classification as the diagnostic criteria for AML, we additionally reviewed and summarized the cohort according to the WHO 2022 classification to provide updated disease classification information.

Association between first-line Aza/Ven response and overall survival should be done according to ELN-24 risk groups.

Response: Thank you for this valuable suggestion. In response to the reviewer’s comment, we reclassified the study cohort according to the recently proposed ELN 2024 genetic risk classification for patients with newly diagnosed AML receiving less-intensive HMA-based therapies. Because ivosidenib plus azacitidine was not used in patients with IDH1-mutated AML in our cohort and DDX41 was not included in our genomic panel testing, this classification could not be fully applied in a strict sense. Nevertheless, we performed a provisional classification based on the available genetic information.

We also compared patient characteristics stratified by CR/CRi and nonCR with the ELN 2024 Less-Intensive classification and added these results to the revised Table 2 (page 19). In this exploratory analysis, the ELN 2024 Less-Intensive classification was associated with overall survival, whereas no statistically significant difference was observed in CR/CRi rates among the risk groups, although remission rates tended to decrease from favorable to adverse risk. We have added these findings to the revised Results (page 19, Table 2) and Discussion sections (page 31, lines 466-473).

Detail median and range of total AZA-Ven cycles administered.

Response: Thank you for this helpful suggestion. We added the median and range of the total number of Aza/Ven cycles administered to the revised Results section (page14, line 218).

The predictive modeling (LDA/SVM with LOOCV) demonstrates high performance (AUC 0.914), but there is no external validation cohort. Given the small dataset and use of LOOCV, the model is at risk of overfitting. Validation in an independent cohort is essential before clinical applicability and should be highly desirable.

Response: Thank you for this important comment. We agree that the predictive model may be at risk of overfitting because of the relatively small dataset and the use of LOOCV without an external validation cohort. Although obtaining an independent external cohort with harmonized genomic and clinical data for patients treated with Aza/Ven is challenging, we recognize that external validation is essential before this model can be applied clinically. We have therefore emphasized this point as a limitation in the revised Discussion (pages 34-35, lnes 521-529).

Patients across first-line, second-line, and later treatment settings are combined in several analyses. Although the authors justify this approach, the biological and clinical heterogeneity may confound interpretation of results, particularly for modeling analyses.

Response: Thank you for this important comment. We agree that combining patients treated with Aza/Ven in first-line, second-line, and later-line settings may introduce biological and clinical heterogeneity and potentially confound the interpretation of the results, particularly in modeling analyses. To address this concern, we additionally evaluated patients who received Aza/Ven as second-line or later therapy and had NGS performed immediately before Aza/Ven treatment. Because NGS was performed immediately before Aza/Ven in only 13 such cases, the analysis was limited by the small sample size. Nevertheless, a similar trend was observed: the CR rate was 75% in Type 1 cases and 0% in Type 2 cases. These findings suggest that the observed association may also be present in later-line settings. We added the information in the revised manuscript (pages 22-23, lines 332-335).

Some figures (e.g., mutation distribution and circos plots) are difficult to interpret without more detailed legends.

Response: Thank you for this helpful comment. We agree that several figures, including the mutation distribution plot and circos/chord diagram, required more detailed explanations to improve interpretability. Accordingly, we revised the figure legends to provide clearer descriptions of the gene categories, color coding, and the meaning of the connecting ribbons. For Figure 3A, we added the following detailed legend (pages 24-25, lines 347-353). We also modified Figure 2C to demonstrate the classification of each gene more clearly and revised the corresponding legend (page 17, lines 272-273).

Consider simplifying or annotating key findings more clearly.

Response: Thank you for this helpful suggestion. Following the reviewer’s advice, we revised the manuscript, figures, and figure legends to improve clarity and more clearly highlight the key findings, including mutation categories, response differences, and statistical comparisons.

The manuscript alternates between “CR/CRi” and “CR/CR.” Terminology should be standardized throughout.

Response: Thank you for your helpful suggestion. We corrected the typo and standardized the notation to CR/CRi (page 10, lines 179).

While the modeling methodology is well described, the clinical interpretability of the model coefficients could be further emphasized in the main text rather than supplementary materials.

Thank you for this helpful comment. We agree that the clinical interpretability of the model coefficients should be emphasized in the main text. Therefore, while retaining the detailed mathematical description of the modeling approach in the Supplementary Methods, we added an explanation of how the LDA coefficients should be interpreted clinically in the revised Methods and Results section (pages 11, lines 180-181, 183-188, 193-194; pages 27-28, lines 390, 392-419). Specifically, because CR/CRi was defined as the positive class, positive coefficients indicate variables that shift the discriminant score toward predicted CR/CRi, whereas negative coefficients indicate variables that shift it toward predicted non-CR. We also clarified that these coefficients represent model-derived weights within the multivariable classifier and should not be interpreted as causal effects.

Important clinical factors such as performance status, comorbidities, and treatment dosing are not included. The absence of these variables should be acknowledged.

Response: Thank you for this important comment. We agree that clinical factors such as performance status, comorbidities, and treatment dosing may influence treatment outcomes and should ideally be considered. However, complete information on these variables was not available for all patients, and they could not be incorporated into the present analysis. We have acknowledged this limitation in the revised Discussion (page 35, lines 537-545).

The manuscript focuses on efficacy but does not address adverse events or tolerability, which are important for clinical interpretation.

Response: Thank you for this valuable suggestion. We agree that adverse events and tolerability are important for the clinical interpretation of Aza/Ven therapy. However, the primary focus of this study was to evaluate the association between gene mutation patterns and treatment efficacy, including remission and survival outcomes. Because detailed adverse event data were not systematically collected in this analysis, we could not comprehensively assess safety or tolerability. We have added this point as a limitation in the revised Discussion (page 35, lines 537-545).

Reviewer #2: In this study, the authors analyzed patients with AML treated with azacitidine/venetoclax (Aza/Ven) in the prospective HM-SCREEN-Japan 02 study using targeted NGS of 53 genes, cytogenetic analyses, and clinical variables. They demonstrated that mutations classified as type 1 and type 2 were associated with CR rates in patients with AML receiving first-line Aza/Ven therapy. Although the study presents interesting findings, several issues should be addressed.

Response: We appreciate the careful review of our manuscript and suggestions from the reviewer. In order to address your concerns, we have modified our manuscript and figures as follows.

This cohort includes patients with AML treated with Aza/Ven, among whom 46% were classified as AML-MRC. This distribution appears to be skewed compared with that of general AML populations. As both type 1 and type 2 mutations examined in this study are frequently associated with MDS-related AML, the results may be influenced by this cohort composition. Indeed, conflicting effects of type 1 and type 2 mutations on AML treatment outcomes have been reported. The authors should consider analyzing the impact of type 1 and type 2 mutations separately in AML-MRC and non–AML-MRC subgroups.

Response: Thank you for this important comment. We agree that the relatively high proportion of AML-MRC in our cohort may have influenced the observed associations, given that both Type 1 and Type 2 mutations are frequently related to MDS-associated genetic features. Therefore, we performed an additional subgroup analysis stratified by AML-MRC status.

A similar trend was observed when the data were analyzed separately for AML-MRC and non–AML-MRC. Among patients with AML-MRC, the CR rate was 80% in Type 1 cases (4 CR and 1 non-CR) and 27% in Type 2 cases (3 CR and 8 non-CR). Among patients with non–AML-MRC, the CR rate was 69% in Type 1 cases (9 CR and 4 non-CR). Because there was only one Type 2 case among patients with non–AML-MRC, meaningful evaluation of Type 2 in this subgroup was difficult.

We have added these results to the revised manuscript and acknowledged that the limited number of Type 2 cases, particularly in the non–AML-MRC subgroup, precludes definitive conclusions (page 24, lines 335-342).

Differences in baseline characteristics and cytogenetic findings between patients with type 1 and type 2 mutations should be clarified. In addition, the frequencies of individual gene mutations should be presented for both groups. The probability of overall survival (OS) should also be compared between the type 1 and type 2 mutation groups.

Response: Thank you for this constructive comment. In accordance with the reviewer’s suggestion, we added a new table comparing the baseline characteristics and cytogenetic findings between patients with Type 1 and Type 2 mutations (Table 3). We also included the frequencies of individual gene mutations in each group.

In addition, we performed additional statistical comparisons of OS among the four mutation-defined subgroups. OS differed significantly among the Type 1-positive/Type 2-negative, Type 1-negative/Type 2-positive, Type 1-positive/Type 2-positive, and Type 1-negative/Type 2-negative groups (p = 0.0303). In particular, OS was significantly longer in the Type 1-positive/Type 2-negative group than in the Type 1-negative/Type 2-positive group (p = 0.0023). We have added these results to the revised Results section (page 26, lines 378-382) and Figure 3F legend (page 25-26, lines 368-370).

Type 1 and type 2 genes generally correspond to favorable-to-intermediate and intermediate-to-adverse risk categories, respectively, in the ELN 2022/2024 classification. This potential confounding effect should be discussed.

Response: Thank you for this important comment. We agree that the association between Type 1/Type 2 mutations and treatment outcomes may be partly confounded by established genetic risk classifications, because Type 1 genes generally overlap with favorable-to-intermediate risk categories, whereas Type 2 genes tend to overlap with intermediate-to-adverse risk categories in the ELN 2022/2024 classifications. We have added this point to the Discussion as a limitation of the present study (page 35, lines 530-536).

According to the NGS data, 11 patients initially classified as intermediate risk based on cytogenetics alone were reclassified as favorable or adverse risk according to the ELN 2022 risk stratification. The specific genetic abnormalities responsible for this reclassification should be described.

Response: Thank you for this helpful comment. We carefully re-evaluated the ELN 2022 risk classification incorporating NGS data and revised the corresponding description. As a result, we found that 5 patients, rather than 2 patients as stated in the original manuscript, were reclassified from intermediate risk based on cytogenetics alone to favorable risk according to the ELN 2022 risk stratification. All of these cases were reclassified because of the presence of NPM1 mutations. In addition, 9 patients were reclassified from intermediate risk based on cytogenetics alone to adverse risk. The genetic abnormalities responsible for adverse-risk reclassification were EZH2 mutations in 2 patients, RUNX1 mutations in 2 patients, SRSF2 mutations in 2 patients, TP53 mutations in 2 patients, and a U2AF1 mutation in 1 patient. Based on the results we have corrected Figure 2A and clarified the genomic information in the revised Results section (page 16, line 249) and the figure legend (page 17, lines 260-264).

Recently, the ELN 2024 genetic risk classification for patients with newly diagnosed AML receiving less intensive HMA-based therapies (ELN 2024 Less-Intensive) has been proposed. The authors are encouraged to reclassify the study cohort according to this system. Furthermore, patient characteristics (e.g., Table 2), stratified by type 1 and type 2 mutations, should be compared with the ELN 2024 Less-Intensive classification.

Response: Thank you for this valuable suggestion. In response to the reviewer’s comment, we reclassified the study cohort according to the recently proposed ELN 2024 genetic risk classification for patients with newly diagnosed AML receiving less-intensive HMA-based therapies. Because ivosidenib plus azacitidine was not used in patients with IDH1-mutated AML in our cohort and DDX41 was not included in our genomic panel testing, this classification could not be fully applied in a strict sense. Nevertheless, we performed a provisional classification based on the available genetic information.

We also compared patient characteristics stratified by CR/CRi and nonCR or Type 1 and Type 2 mutation status with the ELN 2024 Less-Intensive classification and added these results to the revised Table 2 and Table 3. In this exploratory analysis, the ELN 2024 Less-Intensive classification was associated with overall survival, whereas no statistically significant difference was observed in CR/CRi rates among the risk groups, although remission rates tended to decrease from favorable to adverse risk. In addition, We have added these findings to the revised Results (Table 2, Table 3, page 21 lines 309-313) and Discussion sections (page 31, lines 468-475).

The CR rates were reported to be similar across first-line, second-line, and third-line or later Aza/Ven therapy. The number of treatment cycles required to achieve CR in each setting should be provided.

Response: Thank you for this helpful comment. In response to the reviewer’s suggestion, we added the number of treatment cycles required to achieve CR/CRi in each treatment-line setting. In each treatment-line group, including first-line, second-line, and third-line or later therapy, the median number of cycles from Aza/Ven initiation to achievement of CR/CRi was 1 (range, 1–2). This information has been added to the revised Results section (pages 14-15, lines 222-224).

The association between type 1 and type 2 mutations and outcomes following second-line or later Aza/Ven therapy should also be evaluated.

Response: Thank you for this important comment. We agree that combining patients treated with Aza/Ven in first-line, second-line, and later-line settings may introduce biological and clinical heterogeneity and potentially confound the interpretation of the results, particularly in modeling analyses.

To address this concern, we additionally evaluated patients who received Aza/Ven as second-line or later therapy and had NGS performed immediately before Aza/Ven treatment. Because NGS was performed immediately before Aza/Ven in only 13 such cases, the analysis was limited by the small sample size. Nevertheless, a similar trend was observed: the CR rate was 75% in Type 1 cases and 0% in Type 2 cases. These findings suggest that the observed association may also be present in later-line settings. We added the information in the revised manuscript (pages 22-23, lines 332-335).

Although the authors propose a mathematical model integrating baseline clinical variables, even in patients without established prognostic gene mutations, its added value in the context of NGS-based analyses remains unclear. The number of patients included in this model and the 95% confidence interval of the area under the ROC curve should be provided. Furthermore, using the ROC-derived cutoff value, CR rates and OS probabilities could be assessed.

Response: Thank you for this important comment. We agree that the added value and performance of the mathematical model should be more clearly described in the context of NGS-based analyses. In response to the reviewer’s suggestion, we clarified the number of patients included in the model and added the 95% confidence interval for the ROC-AUC.

This modeling analysis included 17 Aza/Ven-treated cases without dominant Type 1 or Type 2 mutations. Under LOOCV, the LDA model achieved an ROC-AUC of 0.914 (95% CI, 0.744–1.000 by DeLong’s method) and an overall accuracy of 88.2%. We also performed an exploratory cutoff-based analysis using the Youden index-derived cutoff for the LOOCV-predicted LDA probability. Using this cutoff, the predicted responder group showed a significantly higher CR/CRi rate than the predicted non-responder group (100.0% [9/9] vs 12.5% [1/8]; Fisher’s exact P = 0.000411).

We further assessed OS using the same cutoff. The predicted responder group showed a numerically higher 12-month OS probability than the predicted non-responder group (77.8% vs 60.0%); however, this difference was not statistically significant (log-rank P = 0.445). Because the cutoff-defined groups were almost identical to the observed response categories and the number of evaluable cases was limited, we interpreted this OS analysis as exploratory rather than as independent prognostic validation of the model. These results have been added to the revised Results section (pages 27-28, lines 390, 392-419).

In the legend of Figure 1c, is “CRR by Aza/Ven line of therapy (Chemotherapy)” correct?

Response: Thank you for kindly pointing that out. We corrected the mistake. (page 15, line 233)

References should be provided for the statements in lines 269–270 and 370–371.

Response: Thank you for the advice. We provided the references (page 18, line 287; page 30, line 454).

The definitions of negative, dim, and positive expression for CD7 and CD56 should be clearly described.

Response: Thank you for this important comment. In this multicenter study, CD7 and CD56 expression levels were assessed by flow cytometry at each participating institution, and the categories of negative, dim, and positive expression were defined according to each institution’s local criteria. We have clarified this point in the revised Methods section (pages 7-8, lines 123-127).

Attachment Submitted filename: Response to Reviewers.docx

# Peer review: Decision Letter 1

**DOI:** 10.1371/journal.pone.0357898.r003

**Authors:** Francesco Bertolini

30 Jun 2026

Dear Dr. Minami,

plosone@plos.org. When you're ready to submit your revision, log on to https://www.editorialmanager.com/pone/ and select the 'Submissions Needing Revision' folder to locate your manuscript file.

- A letter that responds to each point raised by the academic editor and reviewer(s). You should upload this letter as a separate file labeled 'Response to Reviewers'.
- A marked-up copy of your manuscript that highlights changes made to the original version. You should upload this as a separate file labeled 'Revised Manuscript with Track Changes'.
- An unmarked version of your revised paper without tracked changes. You should upload this as a separate file labeled 'Manuscript'.

If you would like to make changes to your financial disclosure, please include your updated statement in your cover letter. Guidelines for resubmitting your figure files are available below the reviewer comments at the end of this letter.

If applicable, we recommend that you deposit your laboratory protocols in protocols.io to enhance the reproducibility of your results. Protocols.io assigns your protocol its own identifier (DOI) so that it can be cited independently in the future. For instructions see: https://journals.plos.org/plosone/s/submission-guidelines#loc-laboratory-protocols. Additionally, PLOS ONE offers an option for publishing peer-reviewed Lab Protocol articles, which describe protocols hosted on protocols.io. Read more information on sharing protocols at https://plos.org/protocols?utm_medium=editorial-email&utm_source=authorletters&utm_campaign=protocols.

As the corresponding author, your ORCID iD is verified in the submission system and will appear in the published article. PLOS supports the use of ORCID, and we encourage all coauthors to register for an ORCID iD and use it as well. Please encourage your coauthors to verify their ORCID iD within the submission system before final acceptance, as unverified ORCID iDs will not appear in the published article. *Only* the individual author can complete the verification step; PLOS staff *cannot* verify ORCID iDs on behalf of authors.

We look forward to receiving your revised manuscript.

Kind regards,

Francesco Bertolini, MD, PhD

Academic Editor

PLOS One

Journal Requirements:

If the reviewer comments include a recommendation to cite specific previously published works, please review and evaluate these publications to determine whether they are relevant and should be cited. There is no requirement to cite these works unless the editor has indicated otherwise.

[Note: HTML markup is below. Please do not edit.]

Reviewers' comments:

Reviewer's Responses to Questions

**Comments to the Author**

Reviewer #1: (No Response)

Reviewer #2: All comments have been addressed

**********

2. Is the manuscript technically sound, and do the data support the conclusions?

Reviewer #1: Partly

Reviewer #2: Yes

**********

3. Has the statistical analysis been performed appropriately and rigorously?

Reviewer #1: Yes

Reviewer #2: Yes

**********

4. Have the authors made all data underlying the findings in their manuscript fully available?

The PLOS Data policy

Reviewer #1: Yes

Reviewer #2: Yes

**********

5. Is the manuscript presented in an intelligible fashion and written in standard English?

Reviewer #1: Yes

Reviewer #2: Yes

**********

Reviewer #1: The authors have satisfactorily addressed the majority of the reviewers' concerns through additional analyses, clarification of the methodology, and a more balanced discussion of the limitations. Only minor revisions are still warranted, mainly to further temper the interpretation of exploratory subgroup analyses and to clarify that the predictive model is not yet ready for clinical implementation without external validation.

Although several additional subgroup analyses have been performed, it remains unclear whether the Type 1/Type 2 mutation classification provides independent predictive information beyond established prognostic factors. If a multivariable model cannot be performed because of the limited sample size, this limitation should be explicitly acknowledgedThese remaining issues do not substantially affect the overall quality of the work.

Reviewer #2: The authors have adequately addressed the reviewer’s comments and provided appropriate revisions throughout the manuscript. The responses are clear and comprehensive, and the revisions have improved the overall quality and clarity of the manuscript. I have no further major concerns regarding this submission.

**********

what does this mean?). If published, this will include your full peer review and any attached files.

If you choose “no”, your identity will remain anonymous but your review may still be made public.

**Do you want your identity to be public for this peer review?** For information about this choice, including consent withdrawal, please see our Privacy Policy

Reviewer #1: No

Reviewer #2: No

**********

[NOTE: If reviewer comments were submitted as an attachment file, they will be attached to this email and accessible via the submission site. Please log into your account, locate the manuscript record, and check for the action link "View Attachments". If this link does not appear, there are no attachment files.]

To ensure your figures meet our technical requirements, please review our figure guidelines: https://journals.plos.org/plosone/s/figures

You may also use PLOS’s free figure tool, NAAS, to help you prepare publication quality figures: https://journals.plos.org/plosone/s/figures#loc-tools-for-figure-preparation.

NAAS will assess whether your figures meet our technical requirements by comparing each figure against our figure specifications.

# Author comment: Author response to Decision Letter 2

**DOI:** 10.1371/journal.pone.0357898.r004

20 Jul 2026

Reviewer #1:

The authors have satisfactorily addressed the majority of the reviewers' concerns through additional analyses, clarification of the methodology, and a more balanced discussion of the limitations. Only minor revisions are still warranted, mainly to further temper the interpretation of exploratory subgroup analyses and to clarify that the predictive model is not yet ready for clinical implementation without external validation. Although several additional subgroup analyses have been performed, it remains unclear whether the Type 1/Type 2 mutation classification provides independent predictive information beyond established prognostic factors. If a multivariable model cannot be performed because of the limited sample size, this limitation should be explicitly acknowledged. These remaining issues do not substantially affect the overall quality of the work.

Response:

Thank you for your thoughtful and constructive comments. We appreciate the reviewer’s positive assessment of the revised manuscript. We agree that the interpretation of the exploratory subgroup analyses should be further tempered and that the predictive model should not be considered ready for clinical implementation without external validation.

In response to this comment, we revised the Discussion to explicitly acknowledge that whether the Type 1/Type 2 mutation classification provides predictive information independent of established prognostic factors, including ELN risk classifications, remains unclear. Because of the limited sample size and the small number of patients in each mutation-defined subgroup, a robust multivariable model adjusting for established prognostic factors could not be performed. We also clarified that the LDA/SVM model should be regarded as exploratory and hypothesis-generating, and that external validation in larger independent cohorts is essential before clinical implementation (pages 35-36, lines 524-541).

Reviewer #2:

The authors have adequately addressed the reviewer’s comments and provided appropriate revisions throughout the manuscript. The responses are clear and comprehensive, and the revisions have improved the overall quality and clarity of the manuscript. I have no further major concerns regarding this submission.

Response:

We sincerely thank the reviewer for the positive evaluation of our revised manuscript. We are pleased that the revisions have addressed the reviewer’s concerns and improved the clarity and quality of the manuscript.

Attachment Submitted filename: 30Jun2026 Response to Reviewers.docx

# Peer review: Decision Letter 2

**DOI:** 10.1371/journal.pone.0357898.r005

**Authors:** Francesco Bertolini

24 Aug 2026

Genomic and Clinical Determinants of Response to Azacitidine plus Venetoclax in Acute Myeloid Leukemia: Results from the HM-SCREEN-Japan 02 Study

PONE-D-26-09315R2

Dear Dr. Minami,

We’re pleased to inform you that your manuscript has been judged scientifically suitable for publication and will be formally accepted for publication once it meets all outstanding technical requirements.

Within one week, you’ll receive an e-mail detailing the required amendments. When these have been addressed, you’ll receive a formal acceptance letter and your manuscript will be scheduled for publication.

An invoice will be generated when your article is formally accepted. Please note, if your institution has a publishing partnership with PLOS and your article meets the relevant criteria, all or part of your publication costs will be covered. Please make sure your user information is up-to-date by logging into Editorial Manager at Editorial Manager® and clicking the ‘Update My Information' link at the top of the page. For questions related to billing, please contact billing support.

If your institution or institutions have a press office, please notify them about your upcoming paper to help maximize its impact. If they’ll be preparing press materials, please inform our press team as soon as possible -- no later than 48 hours after receiving the formal acceptance. Your manuscript will remain under strict press embargo until 2 pm Eastern Time on the date of publication. For more information, please contact onepress@plos.org.

Kind regards,

Francesco Bertolini, MD, PhD

Academic Editor

PLOS One

Additional Editor Comments (optional):

Reviewers' comments:

Reviewer's Responses to Questions

**Comments to the Author**

Reviewer #1: All comments have been addressed

Reviewer #2: All comments have been addressed

**********

2. Is the manuscript technically sound, and do the data support the conclusions?

Reviewer #1: Yes

Reviewer #2: Yes

**********

3. Has the statistical analysis been performed appropriately and rigorously?

Reviewer #1: Yes

Reviewer #2: Yes

**********

4. Have the authors made all data underlying the findings in their manuscript fully available?

The PLOS Data policy

Reviewer #1: Yes

Reviewer #2: Yes

**********

5. Is the manuscript presented in an intelligible fashion and written in standard English?

Reviewer #1: Yes

Reviewer #2: Yes

**********

Reviewer #1: Following the successive rounds of peer review, I confirm that the manuscript has satisfactorily addressed all comments and concerns raised by this referee. The revisions have substantially improved the clarity and overall quality of the work, and no further changes are required at this stage.

Reviewer #2: The authors have adequately addressed the reviewer’s comments and provided appropriate revisions throughout the manuscript. The responses are clear and comprehensive, and the revisions have improved the overall quality and clarity of the manuscript. I have no further major concerns regarding this submission.

**********

what does this mean?). If published, this will include your full peer review and any attached files.

If you choose “no”, your identity will remain anonymous but your review may still be made public.

**Do you want your identity to be public for this peer review?** For information about this choice, including consent withdrawal, please see our Privacy Policy

Reviewer #1: No

Reviewer #2: No

**********

# Editor report: Acceptance letter

**DOI:** 10.1371/journal.pone.0357898.r006

**Authors:** Francesco Bertolini

PONE-D-26-09315R2

PLOS One

Dear Dr. Minami,

I'm pleased to inform you that your manuscript has been deemed suitable for publication in PLOS One. Congratulations! Your manuscript is now being handed over to our production team.

At this stage, our production department will prepare your paper for publication. This includes ensuring the following:

* All references, tables, and figures are properly cited

* All relevant supporting information is included in the manuscript submission,

* There are no issues that prevent the paper from being properly typeset

You will receive further instructions from the production team, including instructions on how to review your proof when it is ready. Please keep in mind that we are working through a large volume of accepted articles, so please give us a few days to review your paper and let you know the next and final steps.

Lastly, if your institution or institutions have a press office, please let them know about your upcoming paper now to help maximize its impact. If they'll be preparing press materials, please inform our press team within the next 48 hours. Your manuscript will remain under strict press embargo until 2 pm Eastern Time on the date of publication. For more information, please contact onepress@plos.org.

You will receive an invoice from PLOS for your publication fee after your manuscript has reached the completed accept phase. If you receive an email requesting payment before acceptance or for any other service, this may be a phishing scheme. Learn how to identify phishing emails and protect your accounts at https://explore.plos.org/phishing.

If we can help with anything else, please email us at customercare@plos.org.

Thank you for submitting your work to PLOS One and supporting open access.

Kind regards,

PLOS One Editorial Office Staff

on behalf of

Dr. Francesco Bertolini

Academic Editor

PLOS One
